# Drug delivery pathways to the central nervous system via the brain glymphatic system circumventing the blood‐brain barrier

**DOI:** 10.1002/EXP.20240036

**Published:** 2024-07-09

**Authors:** Xiang Wang, Yue Yin, Huaijuan Zhou, Bowen Chi, Ling Guan, Pei Li, Jinhua Li, Yilong Wang

**Affiliations:** ^1^ School of Medical Technology Beijing Institute of Technology Beijing China; ^2^ Department of Neurology Beijing Tiantan Hospital Capital Medical University Beijing China; ^3^ Advanced Research Institute of Multidisciplinary Sciences Beijing Institute of Technology Beijing China; ^4^ Center for Advanced Biotechnology and Medicine Rutgers University Piscataway New Jersey USA; ^5^ Beijing Institute of Technology, Zhuhai Beijing Institute of Technology Zhuhai China

**Keywords:** blood‐brain barrier, drug delivery, glymphatic system, immune regulation, intraosseous administration into skull, meningeal lymphatic system, subcutaneous administration near cervical lymph nodes

## Abstract

The blood‐brain barrier (BBB) poses daunting challenges in treating diseases associated with the central nervous system (CNS). Recently, the traditional notion of the absence of the lymphatic system in the brain is evolving. The discovery of the glymphatic system in the brain has stimulated tremendous interest in developing new strategies for the treatment of CNS diseases. Leveraging the glymphatic system for CNS drug delivery may pave a new avenue to circumvent the BBB and achieve efficient drug delivery. The review focuses on the glymphatic system of the brain, discussing potential factors affecting its functions and exploring their connections with the meningeal lymphatic system. Finally, the review provides an overview of the drug delivery methods through the glymphatic system to circumvent BBB and regulate brain immunity. These innovative drug delivery methods may significantly improve drug utilization and create new avenues for the treatment of brain diseases.

## Graphical abstract

The discovery of the glymphatic system has opened up a pathway for drug delivery to the brain by circumventing the blood‐brain barrier, which can effectively improve drug utilization. The methods of drug delivery through the glymphatic system include intrathecal injection, intranasal injection, and delivery through the meningeal lymphatic vessels. The meningeal lymphatic system and glymphatic system provide new developments for the future drug delivery treatment of brain diseases.

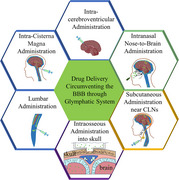

## 1 INTRODUCTION

Central nervous system (CNS) diseases, such as stroke, Alzheimer's, and Parkinson's diseases, are becoming more frequent as global lifespan increases.^[^ ^1^, ^2^ ^]^ Drug delivery to the brain remains the dominant approach for treating CNS diseases. However, the existence of the blood‐brain barrier (BBB) significantly limits the delivery of most drug molecules but a small fraction of uncharged small molecules to the CNS, and even if the BBB is damaged, it is still a daunting challenge for drug molecules passing BBB.^[^ ^3^, ^4^, ^5^, ^6^ ^]^ The BBB is a cellular barrier protecting the brain from endogenous and exogenous toxic substances. Both primary and metastatic glioblastoma are protected by BBB.^[^ ^7^, ^8^ ^]^ Almost all large molecules cannot pass through the BBB, and only some lipophilic small molecules that have molecular weights as low as 400–600 Da can passively penetrate into the brain parenchyma.^[^ ^9^, ^10^, ^11^ ^]^ The BBB greatly limits the delivery of macromolecular drugs to CNS, which is also an important factor affecting the clinical translation of macromolecular drugs.^[^ ^12^, ^13^ ^]^ Therefore, potential new drug delivery pathways across the BBB have drawn broad researchers’ attention.^[^ ^14^, ^15^, ^16^ ^]^ Currently, the known pathways across the BBB include receptor‐mediated endocytosis (transferrin), adsorption‐mediated endocytosis (cell‐penetrating peptides), carrier‐mediated endocytosis (neutrophils, macrophages (Mφ), exosomes, nanoparticles, etc.), and the disruption of BBB integrity to improve the entry of small molecule drugs (magnetic resonance‐guided focused ultrasound: the potential adverse thermal/mechanical effects of ultrasound therapy on healthy tissues limit its clinical application) among many others.^[^ ^16^, ^17^, ^18^, ^19^ ^]^ The BBB‐crossing strategies have been discussed in detail in several reviews.^[^ ^1^, ^20^, ^21^ ^]^ The existence of the BBB remains a major obstacle to efficient CNS drug delivery. The current drug delivery pathway to circumvent the BBB is mainly achieved by the circulation of cerebrospinal fluid (CSF), which accounts for about 10% of the volume of the brain fluid.^[^ ^22^ ^]^ CSF is produced by choroid plexus epithelial cells and circulates outside the brain, thus clearing wastes and metabolites from the brain.^[^ ^23^ ^]^ Maiken Nedergaard's group^[^ ^24^ ^]^ reported that there is a glymphatic system (GS) in the brain composed of perivascular spaces and astrocyte endfeet, which can allow CSF to enter the brain parenchyma and exchange CSF with the interstitial fluid (ISF). Furthermore, it has been confirmed that drugs could enter the GS through CSF, thereby reaching the brain parenchyma for potential disease treatments.^[^ ^24^ ^]^ In addition, the meningeal lymphatic vessels (MLVs) present in the dura mater are connected to the deep cervical lymph nodes (dCLNs) and play a role in clearing the transport of large molecules and immune cells in the brain.^[^ ^25^ ^]^ The GS and meningeal lymphatic system can synergistically promote drug delivery and immune effects in the brain.^[^ ^26^, ^27^, ^28^ ^]^

**FIGURE 1:**
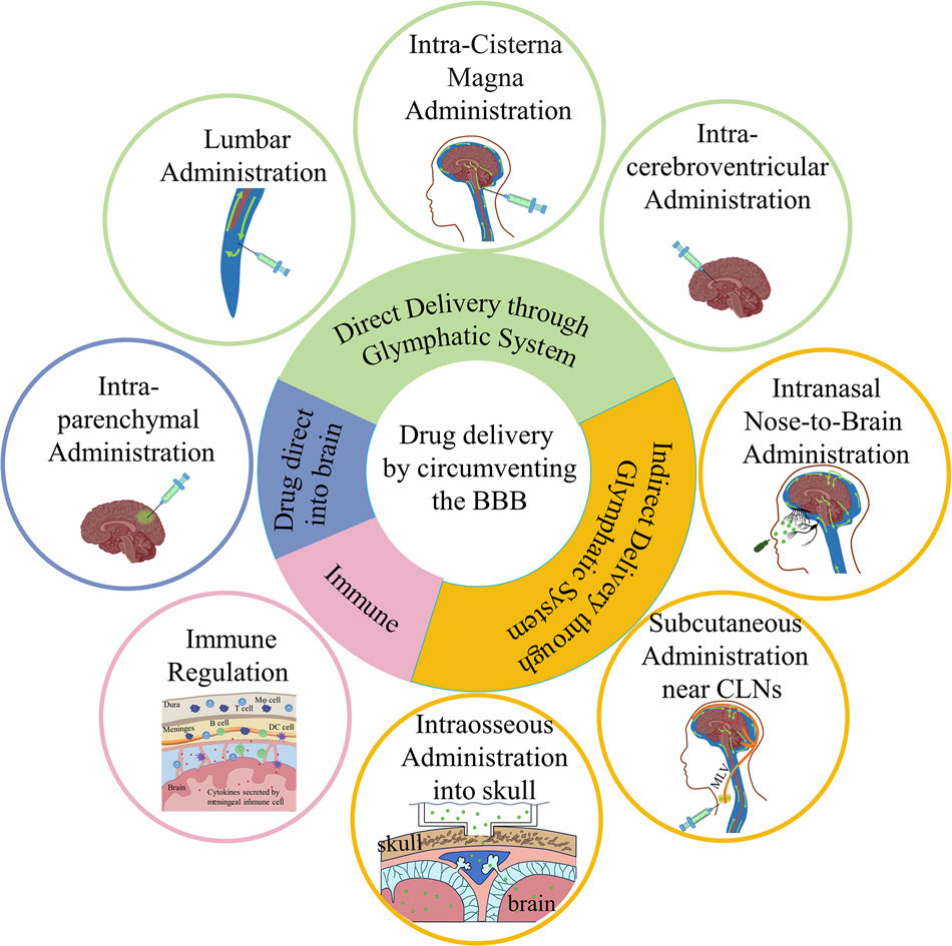
Summary diagram. The drug delivery pathways by circumventing the BBB and immune regulation of the brain lymphatic system. BBB: blood‐brain barrier, CLNs: cervical lymph nodes.

In this review, the importance of the brain's GS and meningeal lymphatic system for innovating CNS drug delivery is highlighted and the effects of aquaporin‐4 (AQP4), sleep, aging, and arterial pulsation on the GS are illustrated. Several potential lymphatic system drug delivery pathways to circumvent the BBB are discussed, including intrathecal administration, nasal administration, subcutaneous administration near cervical lymph nodes (CLN), and intraosseous administration into skull (Figure 1). The role of MLV in brain immunotherapy is highlighted. The review ends by emphasizing the challenges of lymphatic system drug delivery strategies for circumventing the BBB.

## 2 LYMPHATIC SYSTEM OF THE BRAIN

### 2.1 Glymphatic system

**FIGURE 2:**
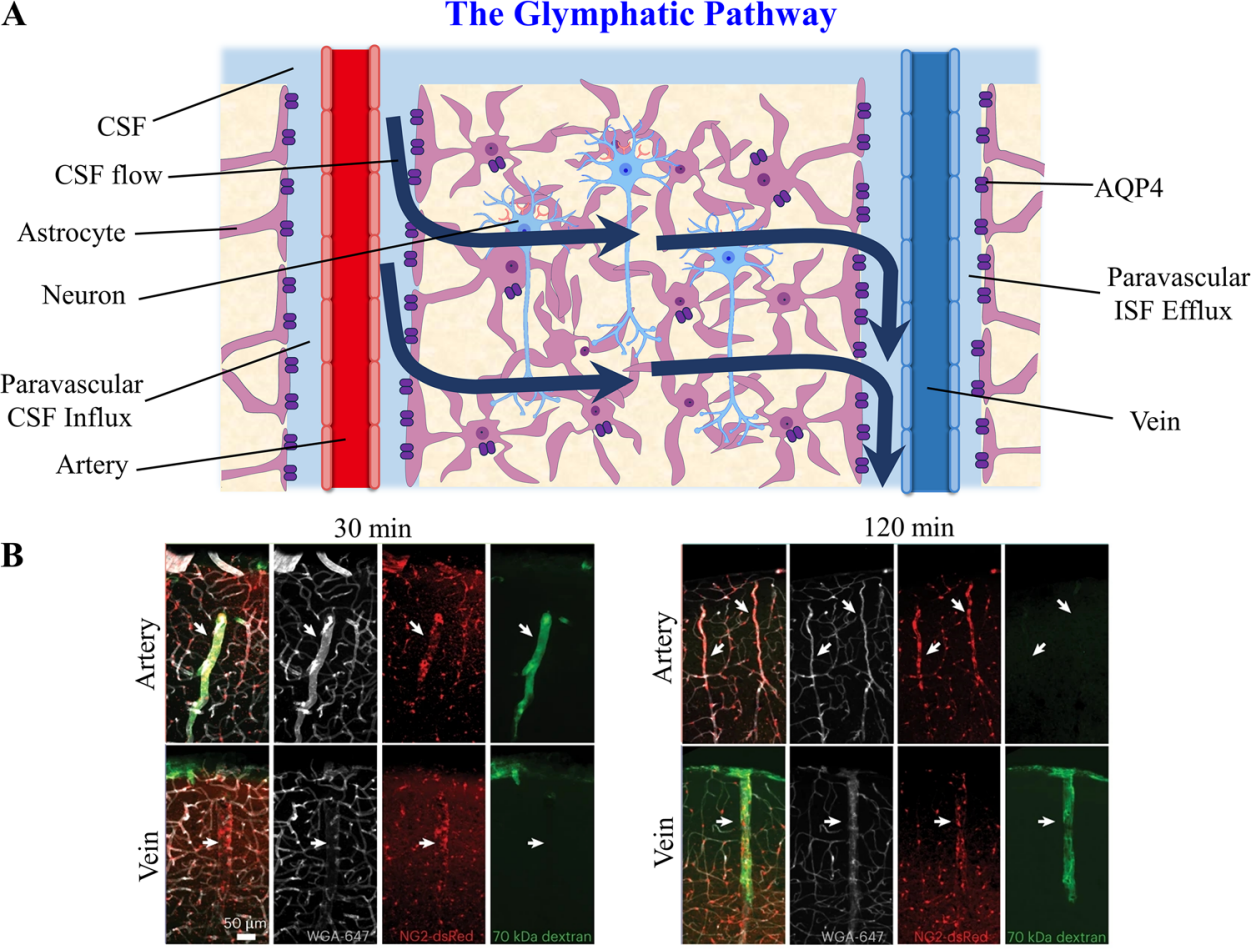
Glymphatic system. (A) The pathways of CSF inflow and outflow within the lymphatic system, as well as the exchange of CSF and ISF. (B) The distribution of different tracers injected into CSF near arteries and veins labeled by NG2‐dsRed. CSF tracers: WGA‐647 (white) and 70 kDa dextran (green). Reproduced with permission.^[^ ^39^ ^]^ Copyright 2023, Springer Nature. CSF: cerebrospinal fluid, AQP4: aquaporin‐4, ISF: interstitial fluid.

The existence of the lymphatic system is crucial for the body's tissue homeostasis.^[^ ^29^ ^]^ However, previous research found a lack of lymphatic vessels in the CNS,^[^ ^30^ ^]^ hence the pathways for clearing metabolites and wastes of the brain demand further studies. The CSF is a solute storage pool outside the brain cells,^[^ ^31^ ^]^ and the ISF in brain tissue constitutes the microenvironment of CNS.^[^ ^32^ ^]^ Studies have found that CSF flows through the ventricles of the brain and eventually returns to the bloodstream, suggesting that waste removal in the brain may be achieved through solute exchange between CSF and ISF, and eventually discharged outside the brain.^[^ ^33^, ^34^ ^]^ Therefore, a thorough study of the circulation of CSF through the brain is needed to determine how the brain clears metabolites and wastes. Iliff et al.^[^ ^24^ ^]^ used an intravenous injection of impermeant fluorescent dextran (CB‐d10) to label arteries and veins, and studied the actual moving direction of CSF in the brain by injecting fluorescent and radiolabeled tracers of different molecular weights into the cisterna magna. Using imaging technology, it was observed that all tracers in the CSF from the subarachnoid space can flow into the brain parenchyma through the paravascular space (PVS: Virchow‐Robin space between the arterial basement membrane and the glia limitans composed by the astrocytic endfeet) of the arteries and are stimulated by arterial pulsation to flow into the brain (Figure 2A).^[^ ^35^ ^]^ Small molecule tracers can also move to the entire brain parenchyma through the pia mater at a fast speed.^[^ ^36^ ^]^ Large molecules are limited to gathering in the PVS, as the perivascular astrocytes can screen for molecular sizes that can enter the brain parenchyma.^[^ ^37^ ^]^ At the same time, they marked the veins connecting the arteries in the brain and injected tracers into the cerebral pool, cortex, or parenchyma. Early experiments confirmed that there was no trace of the tracer around the veins; however, a trace was found next to the veins one hour later, indicating that the CSF and ISF in the brain parenchyma were cleared through the PVS of the cerebral veins.^[^ ^24^ ^]^ Ultimately, a large number of experiments confirmed that CSF flows into the brain parenchyma through the PVS adjacent to the artery, and the size of molecules entering the brain parenchyma is regulated by glial cell boundaries composed of perivascular astrocyte endfeet.^[^ ^38^ ^]^ Subsequently, solute exchange occurs between CSF and ISF, flowing out of the CNS through a paravenous pathway to the jugular vein (Figure 2B).^[^ ^30^, ^39^ ^]^ This paravascular pathway, similar to the lymphatic system, is defined as “glymphatic system”.^[^ ^24^ ^]^ GS has higher permeability than the BBB, opening new potential drug delivery pathways that circumvent the BBB for CNS treatments.

### 2.2 Meningeal lymphatic system

**FIGURE 3:**
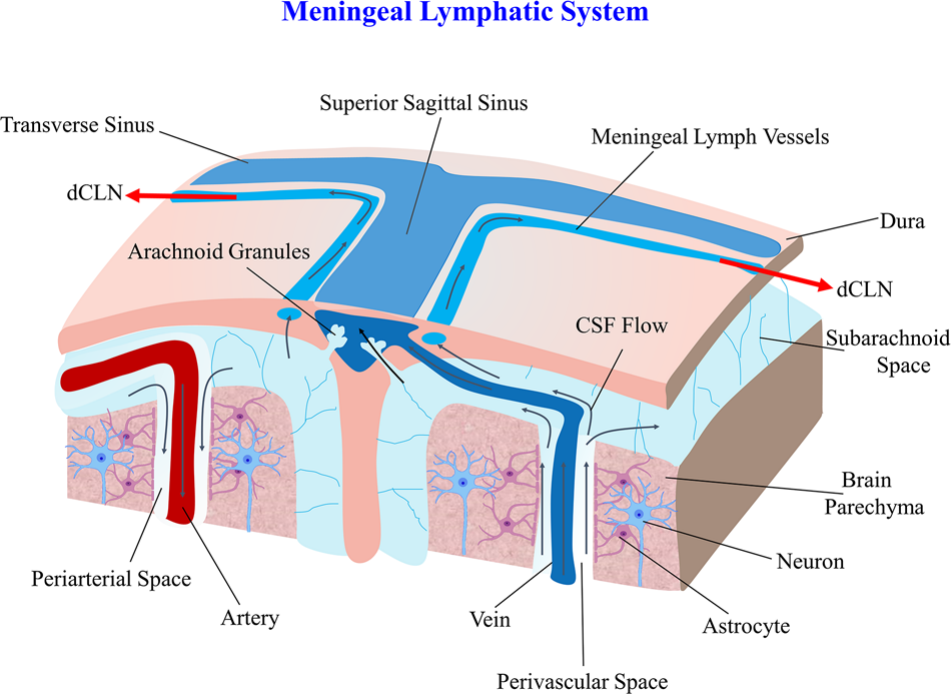
Meningeal lymphatic system. Schematic diagram of the CSF pathways out of the lymphatic system and into dCLN through the MLVs. CSF: cerebrospinal fluid, dCLN: deep cervical lymph nodes.

The discovery of the GS stimulated investigations on new methods for drugs to circumvent the BBB and enter the brain parenchyma through the paravascular pathway. After exchanging with ISF through the GS, CSF flows into the paravenous pathway.^[^ ^24^, ^40^ ^]^ Several studies reported that CSF and metabolites flowing out of the paravascular space of the vein flow out of the brain through certain pathways,^[^ ^40^ ^]^ such as passing through the cribriform plate via the olfactory nerve sheath, flowing along the spinal cord and cranial nerve sheaths into the extracranial lymphatic vessels, entering the venous circulation through arachnoid granules.^[^ ^41^, ^42^, ^43^, ^44^ ^]^ CSF tracers enter the parasagittal dura from the superior sagittal sinus near the entrance of cerebral cortical veins in humans, indicating the presence of trans‐arachnoid molecular channels and suggesting that the parasagittal dura may link the human brain and the dura lymphatic vessels.^[^ ^45^, ^46^ ^]^ However, subsequent experimental studies found that CSF can also be discharged into the CLN, which has generated widespread interest in pathways entering the CLN.^[^ ^47^ ^]^ Further study was conducted on three major meninges in contact with CSF (pia mater, arachnoid membrane, and dura mater), and a rich lymphatic network was ultimately discovered on the dura mater.^[^ ^48^ ^]^ Louveau et al.^[^ ^30^ ^]^ analyzed the anatomical structure and molecular characteristics of the MLV and discovered that MLV lacks smooth muscle cells, positive for immune chemotactic protein CCL21,^[^ ^49^, ^50^ ^]^ exhibits a punctate expresion pattern of molecules and has no expression of integrin‐α9 (a characteristic of lymphatic valves), indicating that the MLV had the characteristics of initial lymphatic vessels.^[^ ^51^ ^]^ To investigate the role of MLV in the pathway of CSF discharge into CLN, Aspelund et al.^[^ ^47^ ^]^ injected a tracer into the mouse brain parenchyma. After 2 h, the tracer was found to flow out of the paravenous space of the vein into the subarachnoid space. A small amount of tracer was also found in the MLV and quickly disappeared, ultimately revealing a strong signal in dCLN. When ligating the efferent lymphatic vessels of dCLN, a large amount of tracer was found in the dural lymphatic vessels. The experiment demonstrated that the MLV absorbs ISF and CSF discharged from the GS through the subarachnoid space, and then drains to dCLN, achieving clearance of fluids and metabolites of brain (Figure 3). It was also found that the absence of MLV had little effect on the clearance of ISF and solutes, implying that there may be other ways to manage fluid outflow. But for the clearance of large molecules in the brain, the dural lymphatic vessels play an important role, such as Alzheimer's disease and other neurodegenerative diseases characterized by misfolded large molecule proteins (amyloid β‐protein, (Aβ)) in the brain.^[^ ^30^ ^]^ The absence of lymphatic vessels in the dura mater may lead to inhibition of the clearance of large molecule proteins.^[^ ^52^ ^]^

### 2.3 Factors affecting the function of the GS

#### 2.3.1 AQP4 activity

**FIGURE 4:**
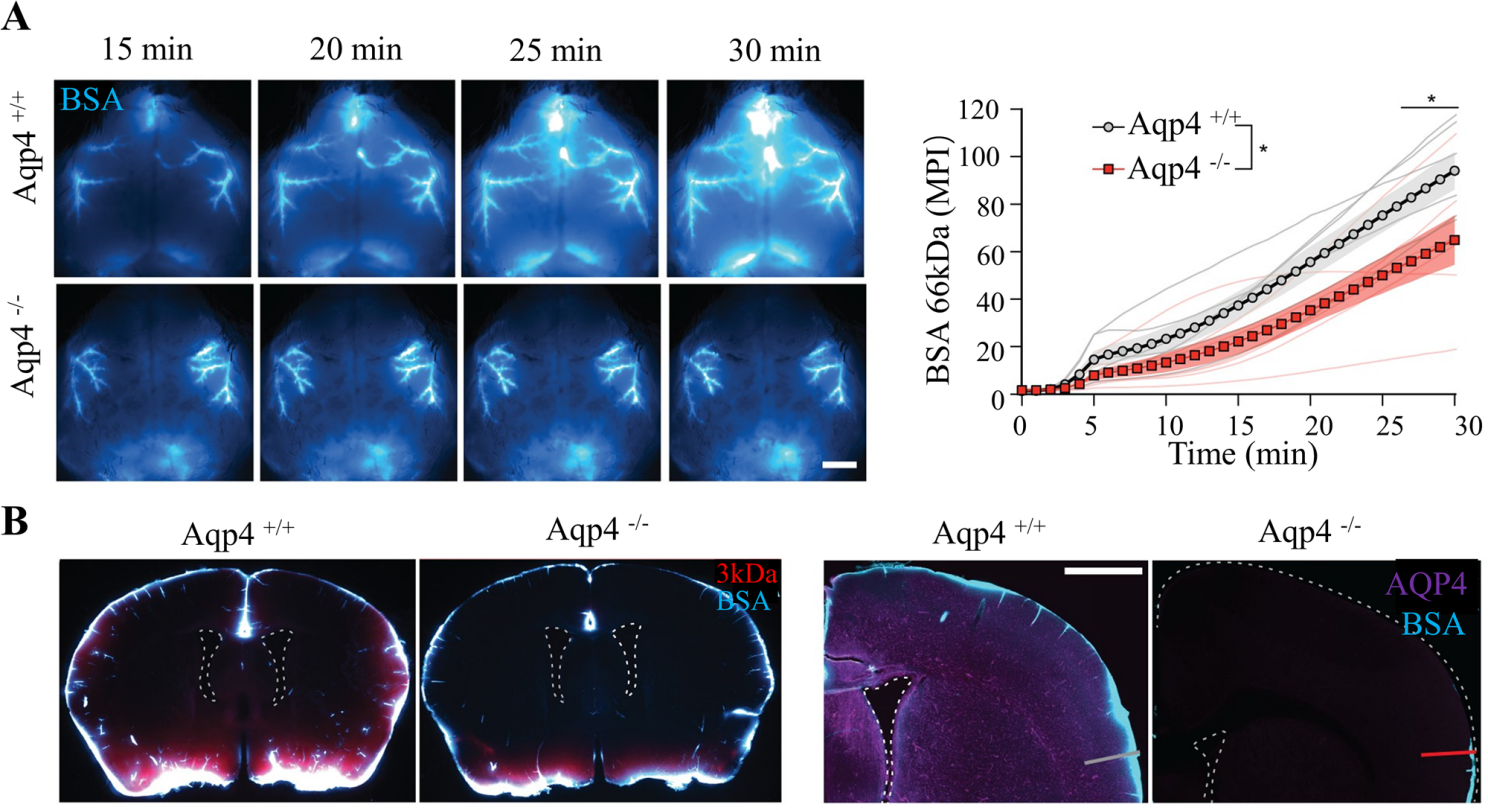
Glymphatic influx of CSF tracer is facilitated by AQP4. (A) Images of transcranial optical imaging experiments of wild‐type control mice (Aqp4^+/+^) and Aqp4 knockout (Aqp4^−/−^) mice injected with BSA and the average pixel intensity within 30 min. (B) Coronary sections were injected with different molecular weight tracers (3 kDa Texas red glucan and BSA‐647) and stained with Aqp4 (magenta). Reproduced with permission.^[^ ^54^ ^]^ Copyright 2018, eLife Sciences. Aqp4: aquaporin‐4, BSA: bovine serum albumin.

AQP4 is an aquaporin present in the CNS, which is highly expressed by astrocytes adjacent to vascular channels and has a bidirectional water transport function.^[^ ^53^ ^]^ AQP4 is located in the membrane perivascular astrocyte end‐feet adjacent to the basal lamina, the inflow of CSF into the GS, and the clearance of ISF depend on AQP4.^[^ ^54^ ^]^ Since the astrocytes next to the PVS only have a ∼20 nm wide gap, the flow of fluid and solutes in the PVS and the brain parenchyma is limited.^[^ ^37^ ^]^ The water movement across glial cells is driven by the static water pressure flowing in para‐arterial pathways, and AQP4 occupies about 50% of the surface area facing the capillaries, leading to low resistance water movement between these compartments, driving solutes into the brain parenchyma.^[^ ^24^ ^]^ Mestre et al.^[^ ^54^ ^]^ injected Alexa 647 conjugated bovine serum albumin (BSA‐647) and a Texas Red 3 kDa dextran into the cisterna magna of anesthetized wild‐type and AQP4 knockout mice (Figure 4). Through in vivo transcranial optical imaging, it was found that CSF entered the brain parenchyma along the para‐arterial pathways, and the absence of AQP4 greatly reduced the flow of both tracers into the brain parenchyma. As time goes by, the differences become increasingly significant (Figure 4A). When different molecular weight tracers were injected, the number of tracers in the brain of AQP4‐deficient mice decreased rapidly. The smaller the molecular weight of the tracer, the easier it is to enter the brain parenchyma (Figure 4B). When the injection volume and rate were adjusted to half of the original, the experiment was consistent with the previous one.^[^ ^54^ ^]^ At the same time, like the inflow pathway of CSF, AQP4 is located near the terminus of venous astrocytes and provides a low‐resistance pathway for water and solute. Research also found the expression of AQP4 near veins is higher than that near arteries, which may contribute to the clearance of brain waste.^[^ ^24^ ^]^

Due to the important role of AQP4 in the GS, regulating its expression has become a new target for maintaining or enhancing the function of the GS.^[^ ^55^ ^]^ Meanwhile, research found that the promotion and inhibition of AQP4 can be beneficial for the treatment of CNS diseases in different situations. For example, inhibiting the function of AQP4 can prevent brain edema caused by acute water intoxication.^[^ ^56^ ^]^ Mestre et al.^[^ ^57^ ^]^ found that a harmful complication after a stroke is cerebral edema, which is closely related to the inflow of CSF. Previous studies have concluded that the GS is an important pathway for CSF to flow into the brain, and this low‐resistance channel can promote the influx of a large amount of CSF, leading to acute tissue swelling. AQP4 is an important mediator that promotes the inflow of CSF.^[^ ^58^ ^]^ Mestre et al.^[^ ^57^ ^]^ did experiments to demonstrate that AQP4‐deficient mice significantly inhibit CSF inflow after embolic middle cerebral artery occlusion, thereby preventing edema after stroke. Misfolded or hyperphosphorylated proteins in the CNS, such as the accumulation of Aβ and tau proteins, are closely related to neurodegenerative diseases, such as Alzheimer's disease; hence the clearance of wrongly accumulated proteins is crucial.^[^ ^59^, ^60^ ^]^ Studies have shown that enhancing the insertion and activity of AQP4 may alleviate or even reverse the progression of neurodegenerative diseases related to proteins.^[^ ^61^ ^]^ However, when AQP4 is wrongly located in the body of astrocytes or loses its polarity distribution on the perivascular astrocyte end‐feet, the clearance rate of proteins through the GS may significantly decrease.^[^ ^62^ ^]^

#### 2.3.2 Sleep

**FIGURE 5:**
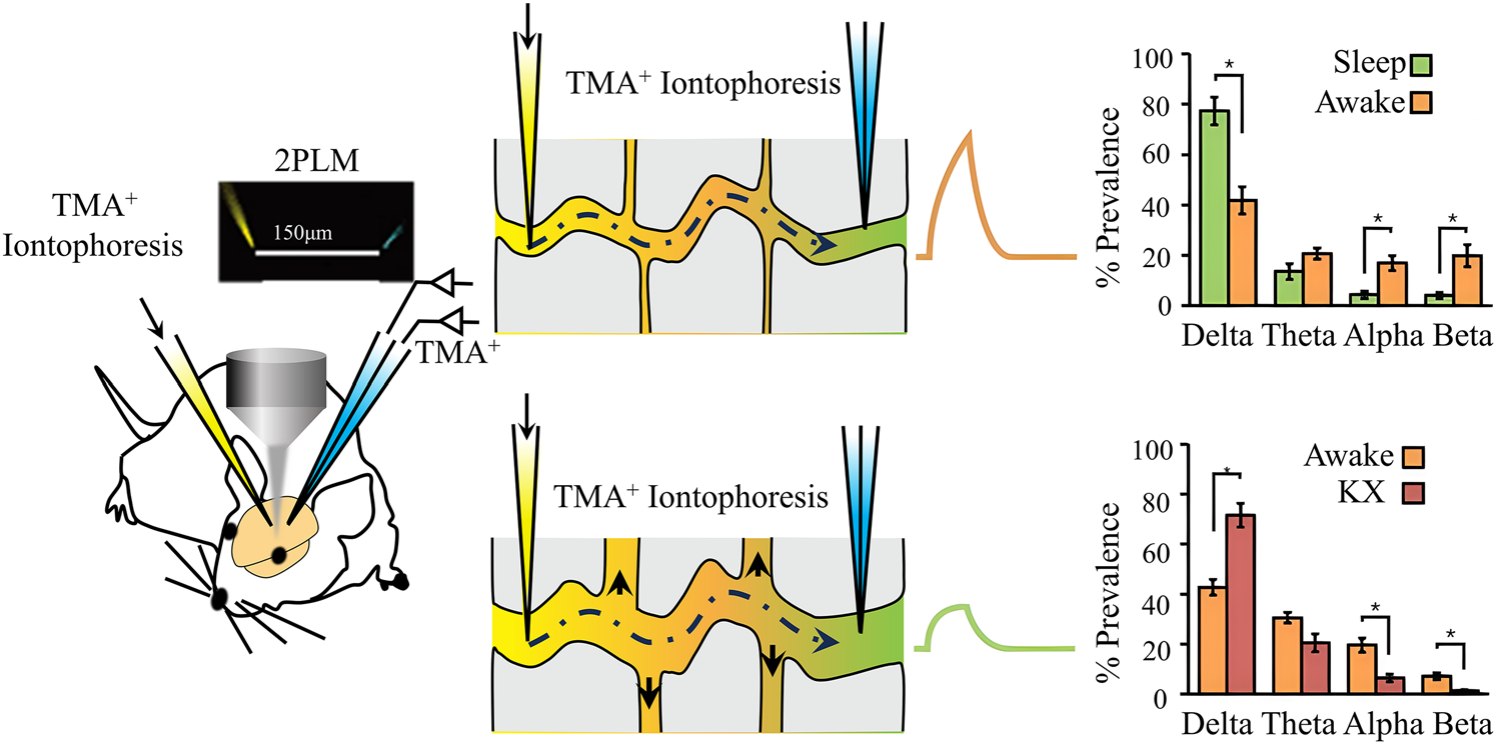
Changes in extracellular space during sleep. By recording TMA^+^ ion electroosmotic quantification using sensitive microelectrodes, the size of extracellular space significantly affects the detected level of TMA^+^. Power spectrum analysis of electrocorticography during waking, sleeping, and anesthesia. Reproduced with permission.^[^ ^69^ ^]^ Copyright 2013, American Association for the Advancement of Science. TMA: tetramethylammonium, KX: ketamine/xylazine anesthesia.

Sleep has been proven to be an important regulator of GS function.^[^ ^63^ ^]^ The inflow of CSF and outflow of cellular metabolites (Aβ, lactate, etc.) of GS are closely related to sleep and sleep cycles,^[^ ^64^, ^65^, ^66^ ^]^ and the relationship is AQP4 dependent.^[^ ^67^, ^68^ ^]^ Xie et al.^[^ ^69^ ^]^ reported that the inflow of CSF and solute clearance are closely related to the volume of interstitial space using the tetramethylammonium (TMA) method. Using fluorescent CSF tracers to study the inflow and solute clearance rate of CSF in mice during sleep and wakefulness, experiments have shown that the inflow and solute clearance rate of sleeping mice (mainly during slow wave sleep) are much higher than those of awake mice, which is beneficial for the flows of CSF and ISF. Xie et al.^[^ ^69^ ^]^ also studied the fluid flow of the GS under anesthesia and concluded that no significant difference compared to the fluid flow during sleep (Figure 5). Although there are significant differences in CSF and ISF flows between the waking and sleeping states, there is no complete closure of the lymphatic system.^[^ ^70^, ^71^ ^]^ The fluid flow of the GS is closely related to the presence and intensity of slow wave activity during sleep. Further experiments found that the inflow of CSF in GS during slow wave sleep significantly increases the clearance rate of CSF and metabolites, while the outflow of CSF and metabolites from CNS significantly increases during wakefulness.^[^ ^72^ ^]^

#### 2.3.3 Aging

**FIGURE 6:**
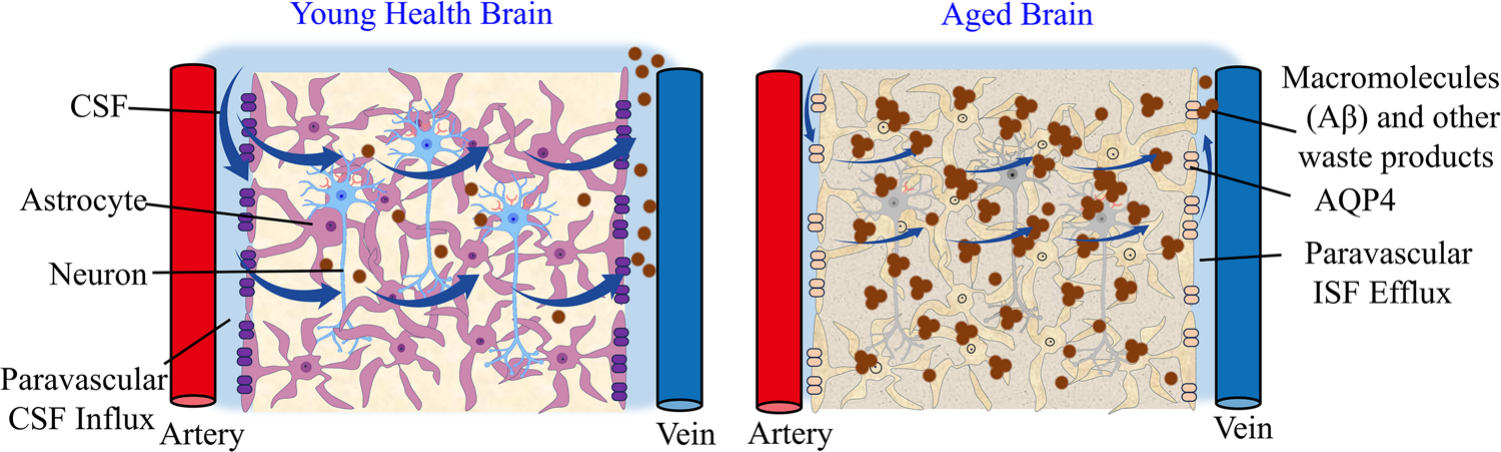
Changes in the GS after aging. Schematic diagram of CSF flow in the GS of the young and aging brains. CSF: cerebrospinal fluid, Aβ: amyloid β‐protein, APQ4: aquaporin‐4, ISF: interstitial fluid.

With the increase of age, the incidence rate of aging‐related central nervous diseases increases significantly.^[^ ^73^ ^]^ Multiple studies hypothesized that aging is related to GS dysfunction (Figure 6).^[^ ^74^, ^75^, ^76^ ^]^ To investigate the relationship between aging and the GS, Kress et al.^[^ ^77^ ^]^ found a significant decrease in interstitial solute clearance in elderly mice and conducted additional studies on the exchange efficiency of CSF and ISF in mice among various age groups. The data showed that the exchange of CSF and ISF in older mice is significantly reduced, suggesting that it may be related to a decrease in the elasticity of cerebral arterioles and AQP4 polarization damage.^[^ ^77^ ^]^ Subsequently, Ma et al. injected tracers into the cisterna magna and ventricles of mice among different age groups, and used imaging techniques to confirm that the CSF inflow and ISF outflow in elderly mice are significantly slower than those in youth mice.^[^ ^78^ ^]^ The above studies indicate that aging is closely related to GS dysfunction, which likely leads to cerebral artery stiffness, reduces pulse amplitude, and results in blockage of perivascular pathways.^[^ ^79^, ^80^ ^]^ Studies also reported that even without the occurrence of diseases and injuries, aging itself is a risk factor for the decline of GS function.^[^ ^81^ ^]^ The clearance efficiency of metabolite decreases in the GS leading to toxic Aβ continuing to accumulate in the brain, triggering the occurrence of CNS diseases.^[^ ^82^ ^]^ Further study is needed on the dysfunction relationship of the age‐related GS. Although aging cannot be avoided, the treatment of age‐related diseases such as Alzheimer's disease and other neurodegenerative diseases may be achieved by improving the function of the GS.^[^ ^83^ ^]^

#### 2.3.4 Arteries pulsation

**FIGURE 7:**
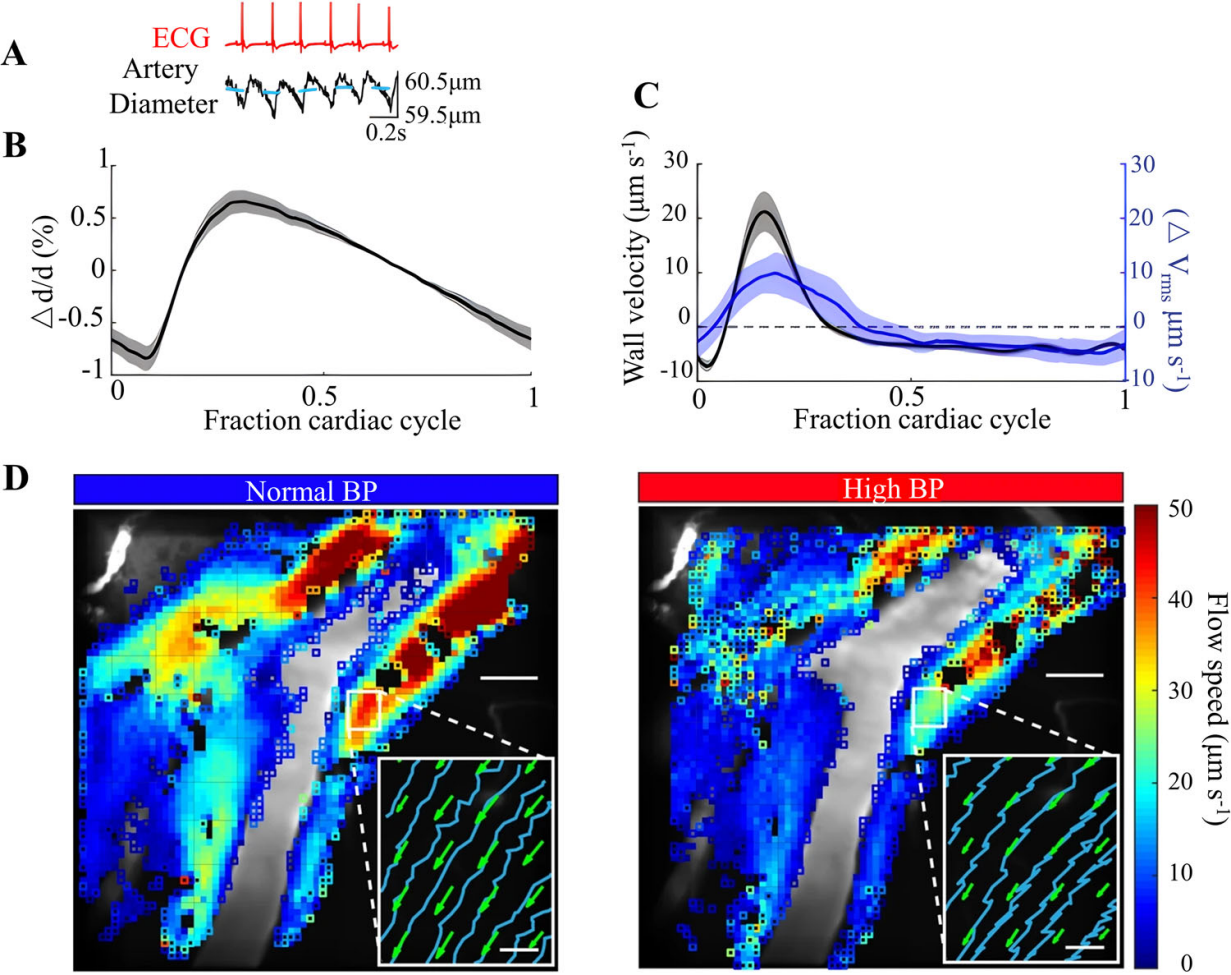
The relationship between arterial pulsation and CSF flow. (A) Simultaneously measured electrocardiogram (red curve), arterial diameter map (black curve), and average arterial diameter (blue dashed line). (B) Changes in arterial diameter during the cardiac cycle. (C) The difference between the arterial wall velocity *v_rms_* (black curve) and its average value during the cardiac cycle is obtained Δ *v_rms_* (blue curve). (D) The CSF flow velocity in the perivascular space under normal blood pressure and hypertension. Illustration: The blue curve represents the trajectory of the microspheres, and the green arrow represents the average flow velocity. It shows an increase in reflux in hypertensive patients. Reproduced with permission.^[^ ^86^ ^]^ Copyright 2018, Springer Nature. ECG: electrocardiogram, BP: blood pressure.

The transportation of liquids and solutes in the GS has been shown to be facilitated by the peristaltic movement of blood vessel walls.^[^ ^76^ ^]^ Further studies confirmed that arterial pulsation has a regulatory effect on the GS.^[^ ^84^ ^]^ Iliff et al.^[^ ^85^ ^]^ observed the pulsation of the blood vessel wall in mice through a two‐photon microscope. Unilateral ligation of the internal carotid artery reduces arterial pulsation, and systemic administration of the adrenergic agonist dobutamine can promote arterial pulsation. At the same time, in vitro fluorescence imaging was used to evaluate CSF influx and CSF‐ISF exchange in GS. Corresponding experiments showed that the rate of CSF inflow and CSF‐ISF exchange decreased after arterial ligation, while the rate of inflow and exchange increased when using the adrenergic agonist dobutamine. The above experiments confirmed that arterial pulsation is an important factor affecting CSF inflow and CSF‐ISF exchange. Mestre et al.^[^ ^86^ ^]^ validated the close correlation between cardiac cycle and CSF flow, and calculated the standardized mean change in arterial diameter during the cardiac cycle by testing electrocardiogram and arterial diameter (Figure 7A). Studies have shown that arterial diameter increases rapidly during ventricular contraction while decreasing slowly during diastole (Figure 7B). The lateral velocity (*v_rms_*) of the arterial wall was calculated and finally concluded that the peak and delay time of the wall *v_rms_* are consistent with the peak and delay time of Δ*v_rms_*, indicating that CSF flow is driven by local displacement of the arterial wall. Arterial pulsation related to respiration can also promote the fluid flow of GS to a certain extent (Figure 7C). Mestre et al.^[^ ^86^ ^]^ also studied the changes in CSF flow around the vascular wall caused by hypertension by changing the local blood pressure of the blood vessels. Research showed that during hypertension, the average flow rate significantly decreases, which may be due to vascular wall sclerosis. The downstream velocity of each component was calculated, and hypertension increased the negative downstream *v_rms_* (reflux), resulting in a decrease in the probability of perivascular pumping (Figure 7D). Overall, CSF flow is driven by arterial pulsation and significantly decreases under hypertension.^[^ ^87^, ^88^ ^]^ Some studies have also shown that aging and CNS disease mice exhibit a decrease in arterial pulsation, leading to a decrease in GS function.^[^ ^77^, ^89^, ^90^, ^91^ ^]^

## 3 DRUG DELIVERY TO THE BRAIN BY CIRCUMVENTING THE BBB

**FIGURE 8:**
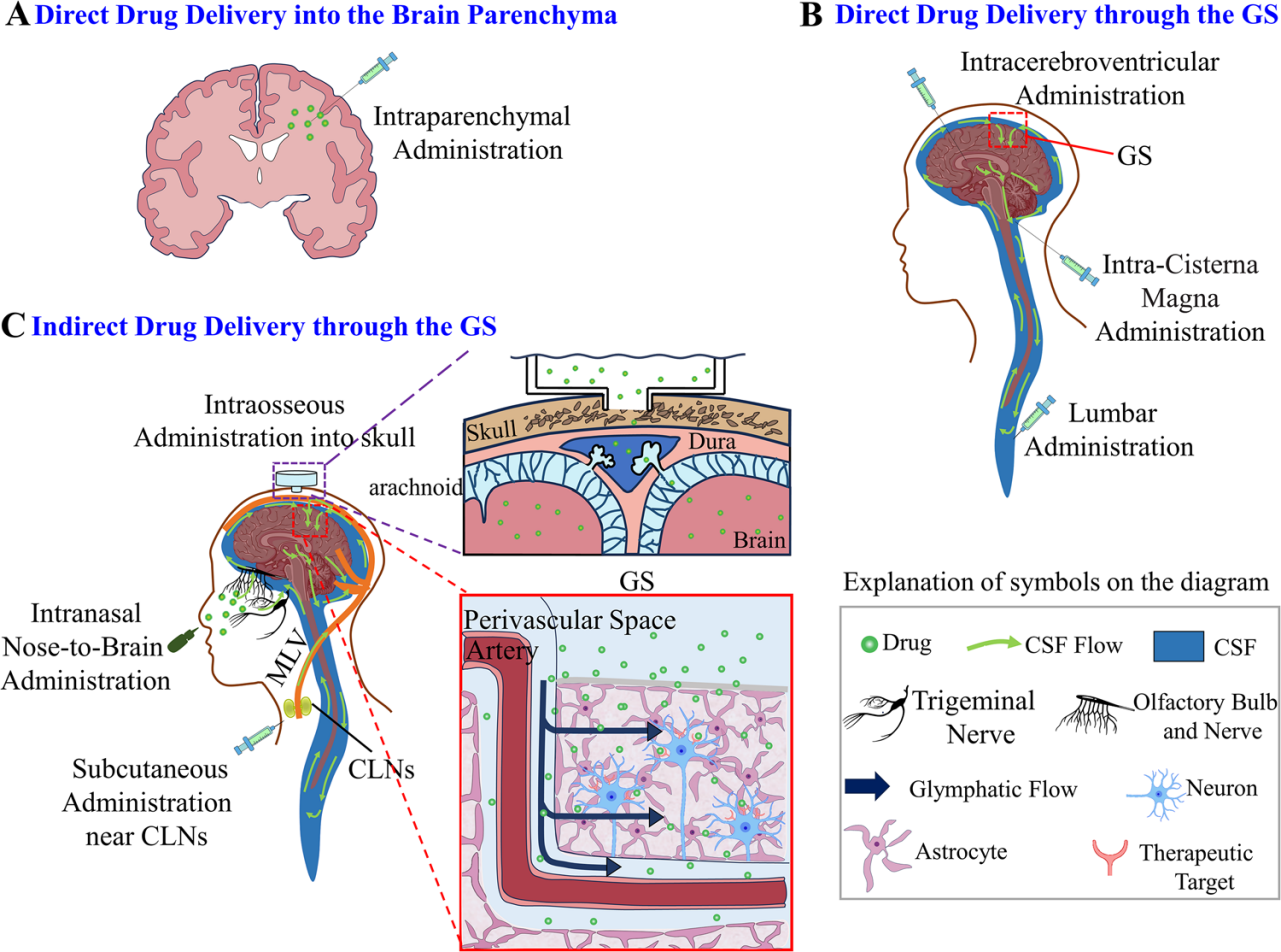
The pathway for drug delivery to the brain. Schematic diagram of (A) intraparenchymal administration, (B) drug administration through intrathecal administration into the CSF, (C) intranasal to brain delivery, subcutaneous administration near the CLNs, and intraosseous administration into skull. GS: glymphatic system, CSF: cerebrospinal fluid, CLN: cervical lymph nodes.

Since the limitation of BBB on the efficiency of drug delivery to CNS, studying drug delivery methods that circumvent BBB has become increasingly important. Many methods have been reported in the open literature to reduce the impact of the BBB, including systemic delivery through the BBB, such as receptor, carrier, and adsorption‐mediated transcytosis, as well as the addition of carriers such as viruses (AAV), cell‐penetrating peptides, exosomes, etc.^[^ ^20^, ^21^ ^]^ The methods for drug delivery to circumvent the BBB involves direct administration into (1) brain parenchyma^[^ ^92^ ^]^ (intraparenchymal administration) (Figure 8A) and (2) CSF (intrathecal administration: lumbar administration, intracisternal magna administration, and intracerebroventricular administration)^[^ ^93^ ^]^ (Figure 8B), and (3) indirect methods (intranasal brain delivery,^[^ ^94^ ^]^ subcutaneous administration near CLN,^[^ ^95^ ^]^ and intraosseous administration into skull (Figure 8C)). Most studies showed that circumventing the BBB for drug delivery is more efficient than blood circulation drug delivery. Here we summarize examples of administration methods that circumvent the BBB.

### 3.1 Intraparenchymal administration

**FIGURE 9:**
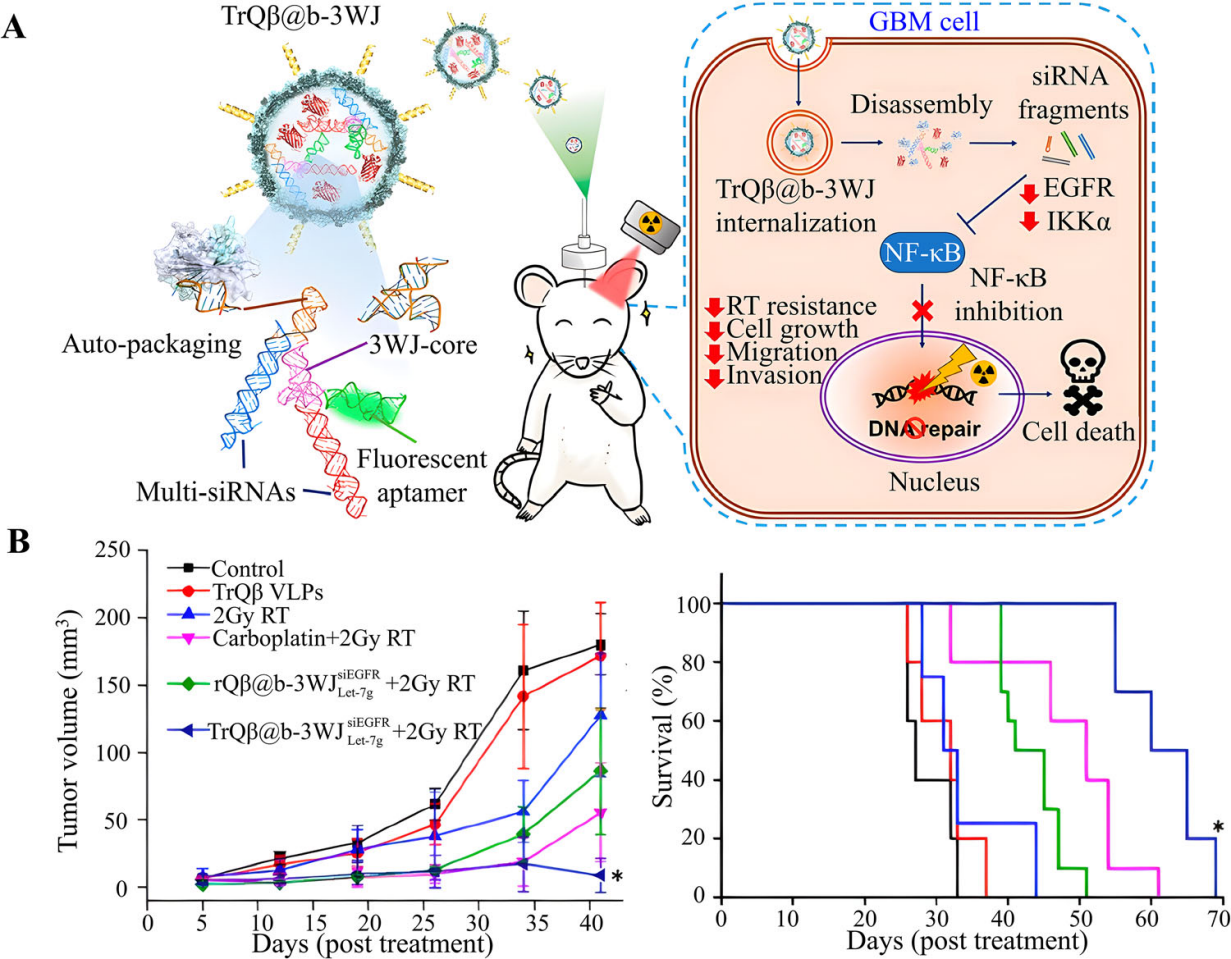
Convection enhanced delivery. (A) Schematic diagram of the effective RT by TrQβ@b‐3WJ using CED infusion. (B) Tumor volume and Kaplan–Meier survival curve of mice after different treatments. Reproduced with permission.^[^ ^103^ ^]^ Copyright 2023, American Chemical Society. GBM: glioblastomas, RT: radiotherapy.

Compared to other drug delivery methods, intraparenchymal administration can more directly affect the CNS, achieve more effective treatment, and reduce corresponding drug losses and side effects.^[^ ^92^, ^96^, ^97^ ^]^ Nance et al.^[^ ^98^ ^]^ developed paclitaxel‐loaded, poly(lactic‐*co*‐glycolic acid)‐*co*‐PEG block copolymer nanoparticles with an average particle size of around 70 nm. Through hydrophilic and uncharged polymer PEG on the surface, the nanoparticles can effectively reduce the adhesion interaction between the nanoparticles and brain parenchyma, enhancing tumor infiltration of nanoparticles. Both in vivo and in vitro experiments showed that the nanoparticle can effectively penetrate the brain parenchyma and achieve uniform drug release, significantly delaying the growth of glioma. Compared with systemic administration of chemotherapy drugs, the flow of ISF in the intraparenchymal can accelerate the absorption of locally administered drugs and increase the time for tumor infiltration, achieving better therapeutic effects.^[^ ^20^, ^98^, ^99^ ^]^ Although preclinical studies have demonstrated the effectiveness of intraparenchymal administration, it still faces challenges in clinical practice, such as the inability to treat CNS‐transmitted diseases and the high invasiveness that may lead to complications.^[^ ^100^, ^101^ ^]^ In order to address the disadvantage of poor diffusion of drugs directly injected into the brain parenchyma, convection‐enhanced delivery (CED) method may improve spatial distribution, utilize pressure gradients and concentration gradients to drive the drug to achieve therapeutic effects in the tumor area, and achieve multiple repeated doses to avoid tumor recurrence.^[^ ^102^, ^103^ ^]^ Pang et al.^[^ ^103^ ^]^ utilized CED infusion bioengineer bacteriophage Qβ particles with designed broccoli light‐up three‐way junction (b‐3WJ) RNA scaffold packaging (TrQβ@b‐3WJ) to circumvent the BBB and reduce systemic toxicity for gene therapy (Figure 9A). This treatment can inhibit DNA repair by downregulating EGFR and IKKα, and inactivating NF‐κB signaling, thereby enhancing chemotherapy. Experimental data showed that this treatment effectively inhibits tumor growth and improves the survival rate of organisms (Figure 9B).^[^ ^103^ ^]^ Catania et al.^[^ ^104^ ^]^ conjugated HA with doxorubicin (DOX) and CpG (a Toll‐like receptor‐9 agonist) respectively, and then combined the two conjugates to generate HA‐DOX+HA‐CpG. HA‐DOX+HA‐CpG can increase the induction of immunogenic cell death in tumor cells, re‐educate pro‐tumoral tumor M2‐like cells into anti‐tumoral M1‐like phenotypes, trigger anti‐tumor CD8^+^T cell responses, and increase the long‐term survival rate of animals.^[^ ^104^ ^]^ CED also faces challenges, including the accurate placement of cannula to prevent reflux and complications (meningitis), optimal infusion rate to maximize drug action without damaging tissue, drug or carrier penetration into cancer cells without being cleared by capillaries, and drug targeting of malignant tumors without damaging normal tissues.^[^ ^105^, ^106^, ^107^ ^]^ Diffuse invasive pontine glioma is the most invasive and deadly CNS tumor in childhood.^[^ ^108^ ^]^ Traditional methods of treatment include radiotherapy (RT) and surgical resection; however, the treatment effect is greatly limited due to the strong infiltration of tumor cells and the ingrained inherent location in the brainstem.^[^ ^109^ ^]^ The application of convective enhanced delivery in diffuse invasive pontine glioma has been widely studied.^[^ ^110^, ^111^ ^]^ When combined with RT/surgical treatment/immunotherapy, CED effectively promotes cancer cell apoptosis and prolongs the survival period of living organisms by providing local high concentrations of drugs.^[^ ^112^, ^113^, ^114^ ^]^

### 3.2 Intrathecal administration

Intrathecal administration refers to injecting drugs into CSF (including lumbar locations, cisterna magna, or intracerebroventricular (or intraventricular)), which can achieve high‐concentration drug delivery and reduce off‐target exposure as well as related toxicity.^[^ ^93^ ^]^ Therefore, intrathecal administration can achieve efficient drug delivery compared to intravenous injection.^[^ ^115^ ^]^

Lumbar administration has the advantages of being fast, having no recovery time, and can be intubated for multiple injections.^[^ ^116^ ^]^ However, lumbar administration occasionally produces epidural and subdural leaks, and the surgical process is painful and requires anesthesia.^[^ ^117^ ^]^ The technique to achieve the injection in special circumstances (obesity or spinal abnormalities) is also challenging.^[^ ^118^ ^]^ Research has shown that high accumulation of anesthesia can lead to neurocognitive impairment in children with chronic diseases.^[^ ^119^ ^]^ To address the disadvantage, repeated lumbar puncture for intrathecal medication treatment is necessary. Clinical trials have shown that patients with acute lymphoblastic leukemia or lymphoma can switch from general anesthesia to lumbar puncture with mild/moderate sedative effects.^[^ ^120^, ^121^ ^]^ With the assistance of ultrasound, the trauma of lumbar puncture can also be reduced to a certain extent, and the number of punctures and surgical time may be reduced.^[^ ^122^ ^]^ The invention of a noninvasive (pencil point) lumbar puncture needle has further optimized lumbar puncture, which can reduce sequelae of epidural puncture, reduce pain, and achieve a high success rate of puncture.^[^ ^123^ ^]^ However, the specific use needs to be determined based on the individual patient's situation.

Cisterna magna administration through the contractile muscles at the back of the neck can deliver drugs to the CSF without disturbing the brain parenchyma.^[^ ^124^ ^]^ However, it may lead to damage to the neck muscles and is difficult to recover.^[^ ^93^ ^]^ Additionally, it is difficult to achieve repeated infusion without the help of a catheter, and it affects CSF movement.^[^ ^124^, ^125^ ^]^ When verifying the existence of lymphatic systems, CSF flow, and establishing CNS disease models, injection into the intracisternal magna is often used, which can quickly fill the CSF with fluorescent colorants and cells.^[^ ^126^, ^127^ ^]^

The lateral ventricle is the main injection site^[^ ^123^ ^]^ and can be administered through the Ommaya reservoir.^[^ ^93^ ^]^ Using the Ommaya reservoir requires scalp shaving, skull drilling, insertion of a catheter, and introduction of a catheter needle into the lateral ventricle.^[^ ^128^ ^]^ Although the entire device can effectively inject drugs into the CSF multiple times, it is invasive and the installation process could be painful.^[^ ^128^ ^]^ The common problems are misplacement, obstruction, and infection, which may lead to complications, usually manifested as fever, headache, and reservoir dysfunction.^[^ ^129^ ^]^ As early as the last century, intracerebroventricular administration of drugs was validated accordingly. Entering the lateral ventricle of the experimental body through a permanent indwelling catheter and injecting renin or angiotensin II into it have been reported to cause thirst and a desire for salt.^[^ ^130^ ^]^ Clinical examples of intracerebroventricular injection, such as using the Ommaya reservoir to insert into the lateral ventricle and administering methotrexate to treat leukemia and childhood embryonic CNS diseases have been reported.^[^ ^131^ ^]^ Intracerebroventricular administration can treat various CNS diseases. Li et al.^[^ ^132^ ^]^ achieved effective suppression of microglia‐mediated neuroinflammation through intracerebroventricular injection of MicroRNA‐210 antagomir, reducing brain damage caused by neonatal hypoxic‐ischemic encephalopathy. Intracerebroventricular injection of Fc‐TWEAK was reported to increase BBB permeability and enhance the production of inflammatory mediators, ultimately leading to neurobehavioral deficits related to lupus.^[^ ^133^ ^]^ Intracerebroventricular injection of calcitonin and prostaglandins can effectively inhibit food intake and gastric acid secretion, acting as CNS regulators. On the contrary, intracerebroventricular injection of indomethacin may hinder the action of calcitonin.^[^ ^134^, ^135^ ^]^ Intracerebroventricular injection of enzymes was the first approved treatment for type 2 neuronal ceroid lipofuscinosis.^[^ ^136^ ^]^ Tărlungeanu et al. conducted a three‐week intracerebroventricular administration of branched‐chain amino acids to improve abnormal behavior in adult autism spectrum disorder and motor dysfunction mice.^[^ ^137^ ^]^

The dosage of drug injection can be determined by the space size and clearance rate of the CSF, and intraventricular administration is more easily distributed throughout the entire CSF compared to lumbar administration; however, the operation is more challenging.^[^ ^138^ ^]^ For example, intraventricular administration produces sufficient CSF distribution for the treatment of meningeal leukemia and meningeal cancer with greater reliability.^[^ ^139^, ^140^ ^]^ Intrathecal administration is superior to intravenous injection in the treatment of certain diseases, such as Gram‐negative meningitis with intrathecal administration of amikacin.^[^ ^141^ ^]^ However, certain situations are not suitable for the intrathecal injection. Some anti‐infective drugs (β‐lactam antibiotics) used to treat CNS infections cannot be injected multiple times, as they are more likely to cause CNS side effects than continuous intravenous infusion.^[^ ^142^ ^]^ Personalized injection volumes and treatment plans based on individual situations need to be optimized.^[^ ^138^ ^]^ Sometimes using intrathecal injection alone may not be a good choice, but combining it with intravenous injection may achieve better results.^[^ ^143^ ^]^ The combination of oral anti‐angiogenic drugs, intravenous bevacizumab, and intraventricular chemotherapy was reported to achieve promising results to treat recurrent embryonic tumors of the CNS in children.^[^ ^144^ ^]^ Intraventricular administration of aminoglycosides combined with intravenous administration of meropenem can treat antibiotic‐resistant immovable rod meningitis.^[^ ^145^ ^]^

**FIGURE 10:**
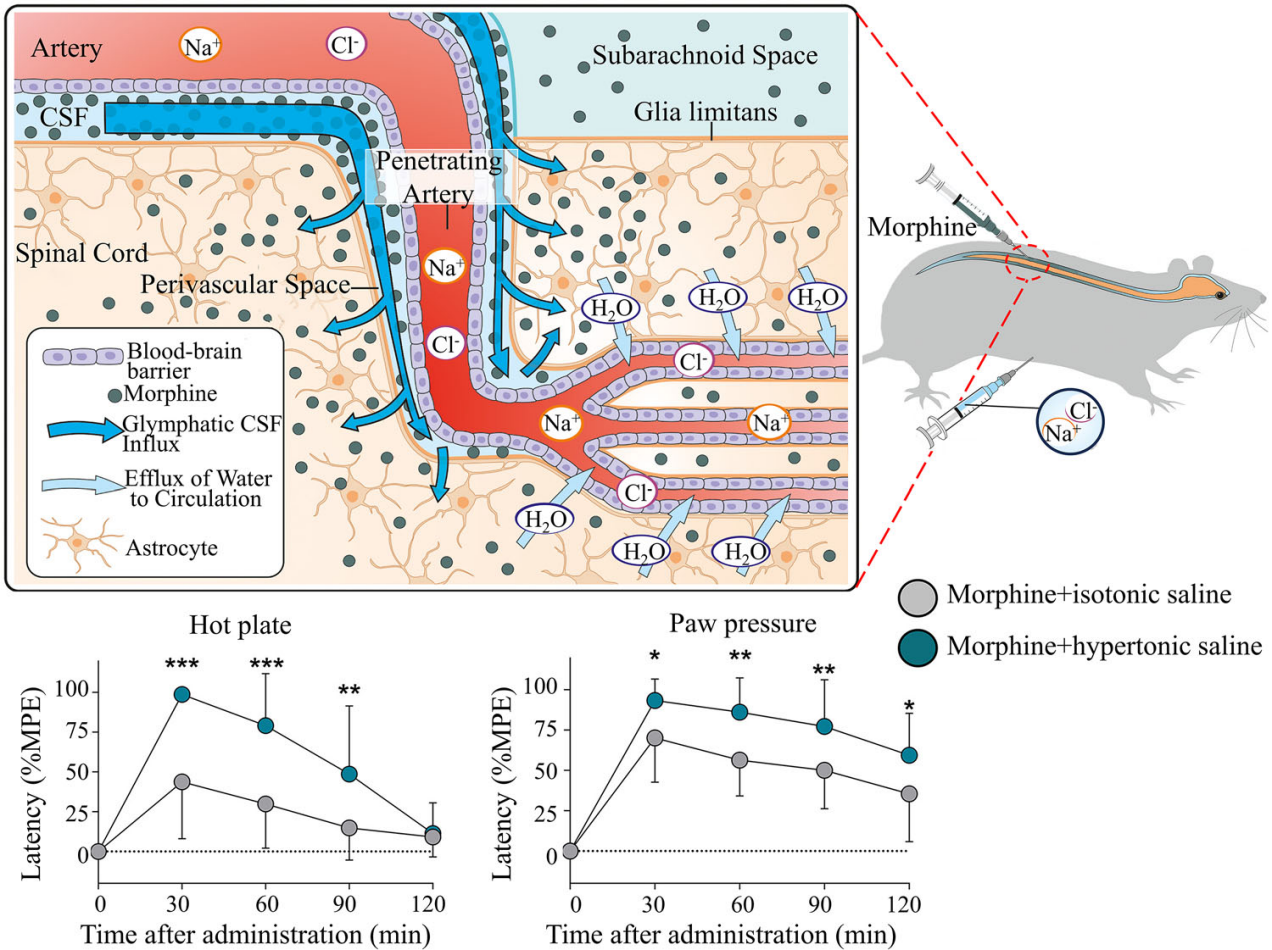
The mechanism of systemic hypertonic saline enhancing the spinal delivery of morphine. The hypertonic saline treatment increased hot plate latencies and the paw pressure thresholds at 30, 60, and 90 min. Reproduced with permission.^[^ ^146^ ^]^ Copyright 2022, Elsevier. CSF: cerebrospinal fluid.

We also concluded that enhancing the inflow of CSF can enhance the absorption of drugs injected into the brain through intrathecal injection. Blomqvist et al.^[^ ^146^ ^]^ evaluated the inflow status of CSF in the hypertonic saline state and the impact of intrathecal injection of morphine on the delivery to the spinal cord (Figure 10). Experiments showed that compared to isotonic saline, systemic injection of hypertonic saline and intrathecally morphine increases the concentration of morphine in the spinal cord and increases the analgesic effect of morphine in the tail flick, hot plate, and paw pressure tests. Similarly, Lilius et al.^[^ ^147^ ^]^ showed through systemic and intrathecal injection of a sedative (dexmedetomidine) that it can enhance the flow of intrathecal drugs from CSF into the CNS.

### 3.3 Intranasal administration

**FIGURE 11:**
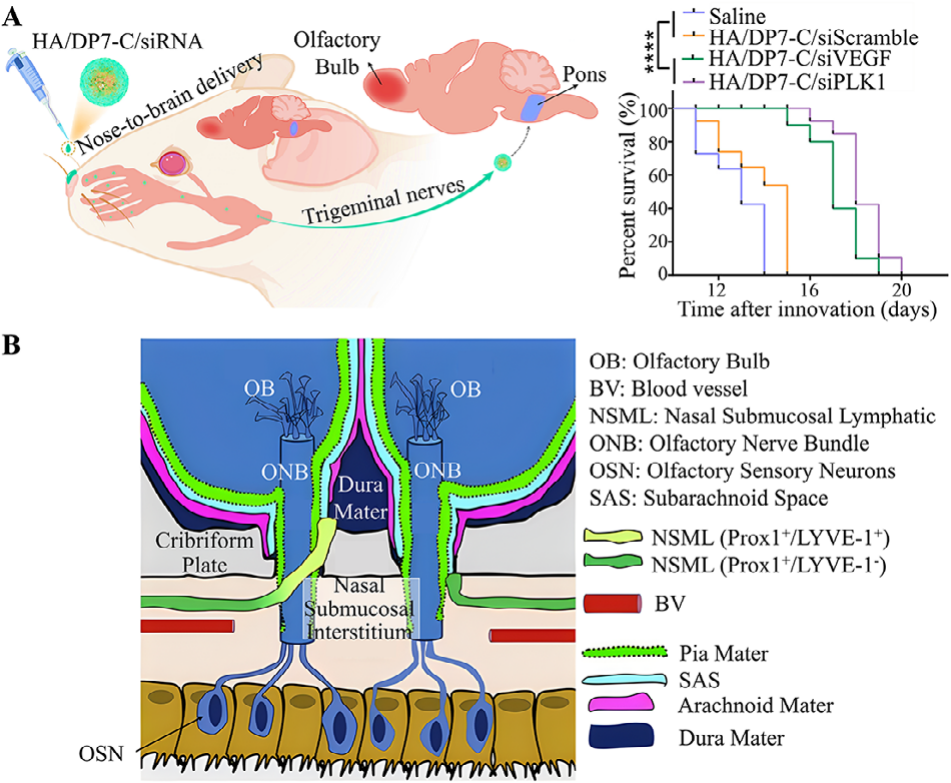
Nose‐to‐brain delivery. (A) Schematic illustration of HA/DP7‐C/siRNA reached the brain after intranasal administration by transport through the trigeminal nerve pathway and the percent survival of GL261 tumor‐bearing mice after different treatments. Reproduced with permission ^[^ ^154^ ^]^. Copyright 2021, Elsevier B.V. (B) The drainage pathway of CSF next to mouse ONB. CSF arrives at SAS from ONB and enters NSML, and finally merges into dCLN. Reproduced with permission.^[^ ^155^ ^]^ Copyright 2023, Elsevier. HA: hyaluronic acid, OB: olfactory bulb, BV: blood vessel, NSML: nasal submucosal lymphatic, ONB: olfactory nerve bundle, OSN: olfactory sensory neurons, SAS: subarachnoid space.

Intranasal administration may enable drugs to be quickly absorbed by the nasal cavity, transmitted through the olfactory and trigeminal pathways to CSF, and enter the brain.^[^ ^148^ ^]^ Kumar et al. used a nasal injection of steroids into the brain in 1977 to effectively prevent ovulation in rhesus monkeys, and the concentration reached in the brain was higher than that of intravenous injection.^[^ ^149^ ^]^ Anatomically, the olfactory bulb and olfactory bulb sensory neurons are located in the cribriform plate, which is located between the nasal cavity and the brain, and CSF flows directly into the brain based on the cribriform plate.^[^ ^150^ ^]^ Drugs can travel along the trigeminal nerve to the brain or be directly transported to the olfactory bulb, bottom of the brain, and perivascular space around cerebral arteries (GS) through intranasal administration, and spread throughout the brain through CSF.^[^ ^151^ ^]^ Nasal drug administration has the advantages of being noninvasive, painless, rapid, and highly manageable by patients. Additionally, compared to oral medications, it can avoid liver metabolism and gastric acid corrosion.^[^ ^152^, ^153^ ^]^ Yang et al.^[^ ^154^ ^]^ prepared multifunctional core–shell structure nano micelles (HA/DP7‐C) using hyaluronic acid (HA) to encapsulate the cell‐penetrating peptide DP7‐C. After intranasal administration, HA/DP7‐C delivered siRNA to the CNS through the trigeminal nerve, successfully achieving the glioma treatment of siRNA (Figure 11A). The pathway of CSF flowing through the olfactory nerve and draining through the cribriform plate to the submucosal lymphatic vessels of the nose and ultimately to the CLNs has been confirmed, which confirmed the feasibility of connecting the brain to the nasal cavity and intranasal administration (Figure 11B).^[^ ^155^ ^]^ Parkinson's disease is primarily characterized by the pathological aggregation of misfolded α‐synuclein (α‐syn) protein.^[^ ^156^ ^]^ The extracellular vesicles in CSF play an important role in α‐syn protein transmission and the olfactory bulb is one of the major sites of transmission.^[^ ^157^ ^]^ Herman and colleagues demonstrated that extracellular vesicles extracted from the CSF of Parkinson's patients can effectively induce the spread of α‐syn from the olfactory bulb to the brain in healthy mice, resulting in Parkinson's‐like syndromes, such as reduced olfaction and motor behavioral disorders.^[^ ^158^ ^]^ In light of this experimental phenomenon, it may be a viable approach to utilize extracellular vesicles for nasal delivery drug to the brain. Although studies have shown the feasibility of intranasal administration, it still faces several obstacles that may lead to poor clinical outcomes.^[^ ^159^ ^]^ For example, the low dosage of self‐administered drugs, the size and performance of molecules are limited by the membrane permeability of the nasal mucosa (small molecule lipophilic drugs are more likely to enter the brain through nasal injection), and the complex anatomical pathway from the nasal cavity to the brain also affects the efficiency of administration.^[^ ^160^ ^]^ In order to improve the low bioavailability of intranasal administration, research suggests that multiple small doses of medication and changing the performance of drug carriers can effectively improve defects.^[^ ^161^ ^]^ The design of the drug delivery device also affects the efficiency of drug delivery to a certain extent. Related studies have been discussed in some reviews.^[^ ^159^ ^]^ Craft et al.^[^ ^162^ ^]^ studied clinical trials in which nasal devices transport insulin into the brain to regulate brain function related to Alzheimer's disease. Preclinical studies confirmed that nasal injection of insulin can circumvent the BBB and enter the brain from the olfactory and trigeminal nerves.^[^ ^163^ ^]^ Clinical experiments have shown that different delivery devices can alter the effectiveness of drugs entering the CNS, showing that delivery devices are equally important for intranasal injection.^[^ ^159^ ^]^

### 3.4 Subcutaneous administration near CLN

The discovery of MLV opens a new possibility for drug delivery to the CNS.^[^ ^47^ ^]^ Several studies proposed new CNS drug delivery strategies regulated by the lymphatic system indicating that the MLV provides a connecting channel between CSF and CLN, which can achieve fluid and immune cell delivery.^[^ ^30^, ^164^ ^]^ Based on this strategy, an increasing number of studies have confirmed that subcutaneous (s.c.) injection of drugs near CLN for delivery to the CNS is promising.

**FIGURE 12:**
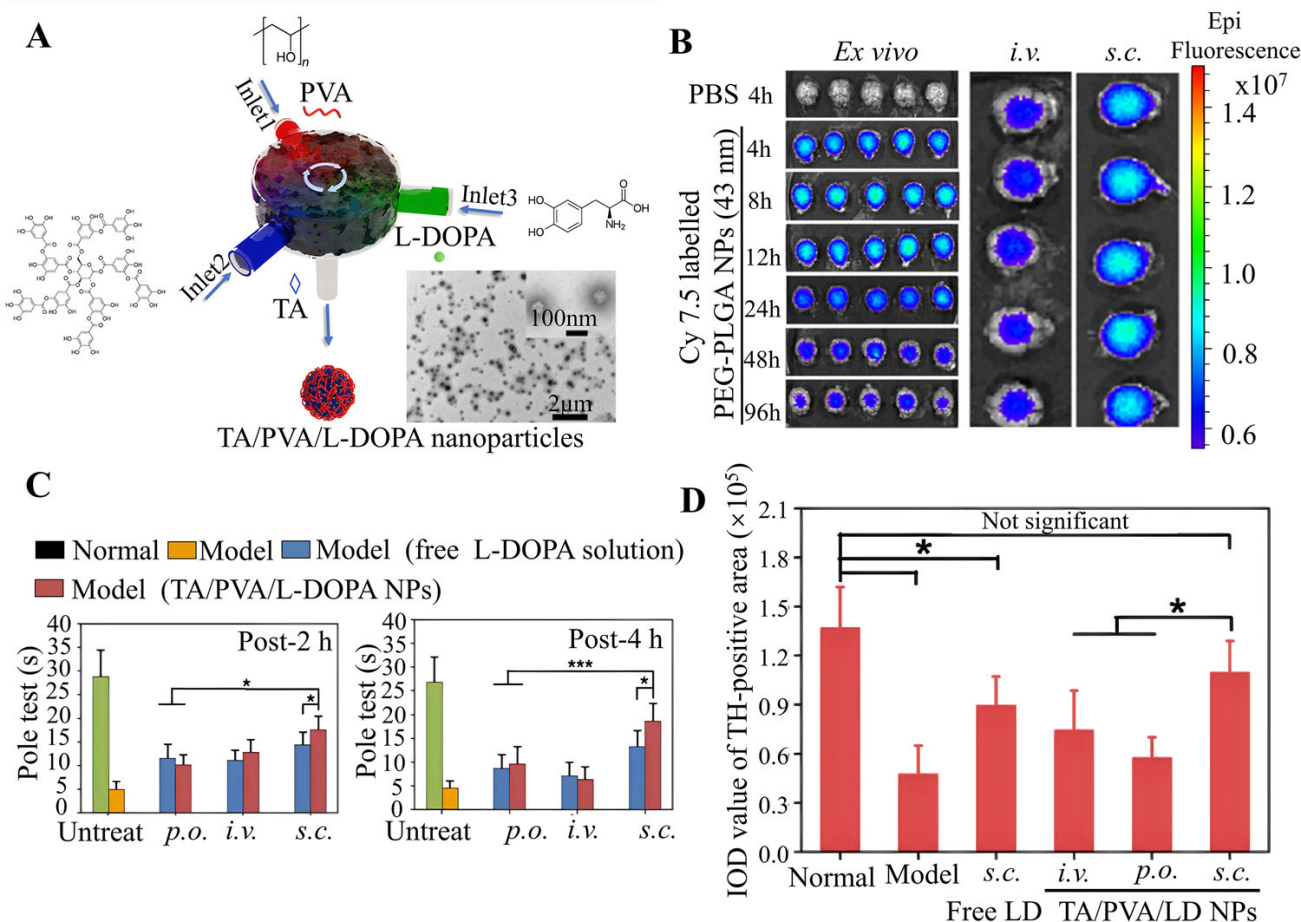
The therapeutic effect after subcutaneous administration near CLN. (A) Schematic diagram and SEM of TA/PVA/L‐DOPA nanoparticles. (B) In vitro fluorescence signal intensity in the brain after injection of drugs. (C) Rod test results after 2 and 4 h of treatment with TA/PVA/L‐DOPA nanoparticles for reserpine‐induced motor dysfunction in a rat model of Parkinson's disease. (D) Semi‐quantitative analysis of IHC results. Reproduced with permission.^[^ ^95^ ^]^ Copyright 2020, Tsinghua University Press and Springer‐Verlag GmbH Germany, part of Springer Nature. TA: tannic acid, PVA: polyvinyl alcohol, LD/ L‐DOPA: levodopa.

Nie et al.^[^ ^95^ ^]^ used a nanocomposite synthesis process to combine levodopa (L‐DOPA), tannic acid (TA), and polyvinyl alcohol (PVA) to generate uniformly sized sub‐50 nm nanoparticles (TA/PVA/L‐DOPA nanoparticles), which were s.c. injected into the neck and transported to the CNS through MLV to treat Parkinson's disease (Figure 12A). The movement disorders of PD rats are mainly due to dopamine deficiency. The use of near‐infrared dye Cy7.5 carboxylic acid for labeling nanoparticles, in conjunction with near‐infrared imaging, can effectively detect the location of the nanoparticles. The experimental results showed that after s.c. injection of the drugs into the neck, the fluorescence signal in the brain gradually became stronger over time. Compared with intravenous (i.v.) administration, it was found that s.c. administration enhanced the fluorescence signal in the brain, demonstrating that it can promote the transport of nanoparticles to the brain parenchyma (Figure 12B). By establishing a Parkinson's disease (PD) model in rats, the improvement of motor disorders was tested by administering TA/PVA/L‐DOPA nanoparticles through three pathways, including oral (p.o.), i.v. administration, and s.c. administration into the neck. Through comparison, it was found that neck s.c. administration had significantly better effects than the other two administration methods in pole holding, suspension, and rod rotation tests, indicating that neck s.c. administration can improve motor disorders in PD rats (Figure 12C). The neck s.c. administration could significantly increase dopamine levels in the striatum, thereby improving motor disorders in rats. In order to further evaluate the striatal dysfunction caused by dopamine deficiency, the occurrence of this phenomenon can be clearly observed through TH expression (Figure 12D). Overall the study showed that the drug delivery mediated by MLV and s.c. injection into the neck near lymph nodes has great potential for the treatment of Parkinson's disease.^[^ ^95^ ^]^

Liu et al.^[^ ^165^ ^]^ found that nanomedicines may be internalized by immune cells rich in MLV, thereby reducing drug transport efficiency. Based on the observation, they utilized a natural killer cell membrane to encapsulate curcumin liposome (BLIPO‐CUR) with the ability to eliminate reactive oxygen species and absorb α‐syn to evade the phagocytosis of Mφ, and a drug delivery platform with a size of about 82 nm was prepared. Liu et al.^[^ ^165^ ^]^ validated the effective MLV delivery strategy for PD‐targeted therapy through drug release, active targeting ability, antioxidant stress, anti‐immune phagocytosis, and α‐syn elimination experiments.

The drug transport in the lymphatic system is closely related to the size of the carrier. Studies have shown that particles ranging from 25 to 100 nm are more suitable for drug transport in the lymphatic system.^[^ ^166^ ^]^ If they are too small, they are prone to extravasation, while if they are too large, they are difficult to enter the lymphatic vessels.^[^ ^167^, ^168^ ^]^ Zhao et al.^[^ ^169^ ^]^ prepared PLGA nanoparticles loaded with ICG in three different sizes (45.9 ± 0.9 nm (NP‐1), 113.2 ± 4.1 nm (NP‐2), and 180.9 ± 3.1 nm (NP‐3)). Experimental results showed that the smallest particle had the highest drug delivery efficacy, which also confirmed the size dependence of brain lymphatic drainage. A control experiment was conducted to establish an MLV ligation mouse model by surgical dissection and ligation of lymphatic vessels. After s.c. neck administration of normal mice and MLV ligation mice, a comparative experiment was conducted to show that the particles were delivered to the brain through the cerebral lymphatic vessels. This study demonstrates that s.c. administration near CLN can effectively deliver drugs to the brain through the MLV pathway, and photodynamic therapy can effectively inhibit the growth of glioblastoma in situ.

Liu et al.^[^ ^170^ ^]^ first developed a disulfide‐lenalidomide‐methoxy polyethylene glycol (LND‐DSDA‐mPEG) nanoprodrug and synthesized an amphiphilic nanoparticle with an average particle size of 58 nm by loading methotrexate (MTX@LND NP). After injection of the nanoparticles into the neck, nanoparticles can be targeted and delivered to brain tissue through the MLV and GS.

### 3.5 Intraosseous administration into skull

**FIGURE 13:**
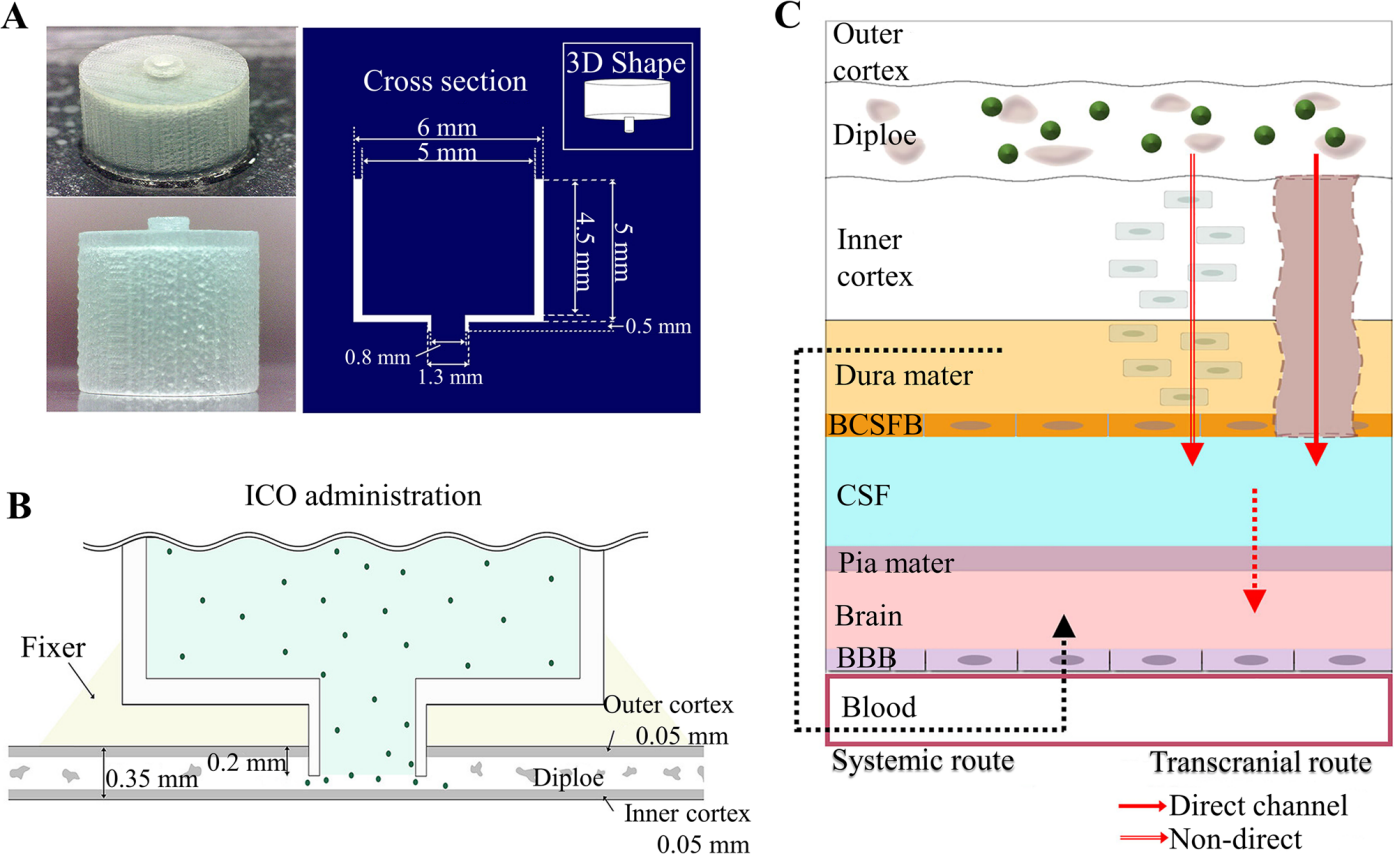
The intraosseous administration of CNS drugs into the skull (ICO) as a novel approach for brain drug delivery via BBB‐bypassing routes. (A) The physical picture and dimensions of the ICO device. (B) Schematic diagram of the drug delivery pathway of ICO administration. (C) Direct and indirect pathways for molecule entry into the brain after ICO administration. Reproduced with permission.^[^ ^175^ ^]^ Copyright 2022, Wiley Periodicals LLC. ICO: intracalvariosseous, CSF: cerebrospinal fluid, BCSFB: blood‐cerebrospinal fluid, BBB: blood–brain barrier.

Research has shown that the skull bone marrow can connect to the dura mater through ossified vascular channels, and CSF can also enter the skull bone marrow through the dura mater, proving direct communication between CSF and the skull bone marrow.^[^ ^171^ ^]^ Acute lymphoblastic leukemia cells tend to metastasize to the CNS, but do not pass through the BBB. Instead, they move along the surface of blood vessels (cavities) connected to the skull bone marrow and subarachnoid space, reaching the CSF and ultimately transferring to the CNS.^[^ ^172^ ^]^ Similarly, meningeal B cells originate from the skull and migrate to the meninges using a nonsystemic channel network (specialized vascular channels within the skull).^[^ ^173^ ^]^ The porosity of the skull allows for passive diffusion of small molecules (≤40,000 MW).^[^ ^174^ ^]^ Roth et al.^[^ ^174^ ^]^ thinned the mouse skull bone to a thickness of ∼20–30 µm and administered the drug through the skull. They found that the drug leaked from blood vessels to the subarachnoid space and surrounding blood vessels, and finally reached parenchymal cells, achieving the treatment of a new type of mild brain injury. Kang et al.^[^ ^175^ ^]^ first proposed intraosseous administration of drugs into the skull (intracalvariosseous (ICO)) as a brain administration method to circumvent the BBB. They also designed ICO devices specifically for this method (Figure 13A). Firstly, the mouse skull was thinned, and then the device was inserted into the skull (Figure 13B). The administration rate of the ICO device was tested using nine compounds. The experimental results indicate that drugs can reach the brain through the skull, and compared to systemic administration, the concentration of drugs in the brain administered by ICO can reach several tens or even hundreds of times (Figure 13C).^[^ ^175^ ^]^ ICO, as a novel approach, has entered our sight. Although it requires thinning of the skull and device installation, as well as more detailed research, it has also opened up a new chapter for brain drug delivery methods that circumvent the BBB.

### 3.6 Conclusion

In conclusion, the drug is delivered to the brain through the lymphatic system mainly by direct or indirect injecting the drug into the CSF (such as intrathecal and intranasal administration), and then utilizing the flow of CSF to enter the GS, ultimately achieving the entire drug delivery pathway to the brain. Secondly, the subarachnoid space absorbs the ISF and CSF expelled by the GS. These fluids then pass through the MLV and are drained into the dCLN, achieving the clearance of liquids and metabolites downstream of the GS. Therefore, when drugs are administered subcutaneously near the CLN or via intraosseous delivery, they can pass through the MLVs, then through the arachnoid mater, to enter the GS, and ultimately reach the brain.

Since most methods to circumvent the BBB involve delivery through the GS to brain tissue, enhancing the function of the GS (increasing lymphatic permeability) can facilitate the efficiency of drug delivery. In the previous sections, we have already discussed factors that affect the function of the GS, such as AQP4 activity, sleep, arterial pulsation, and aging. Therefore, improving the function of the GS can be achieved by regulating the expression of AQP4,^[^ ^55^ ^]^ sleep/anesthesia,^[^ ^69^, ^147^ ^]^ and promoting the production of CSF.^[^ ^176^ ^]^ In the section on intrathecal drug delivery, we also summarized that the use of systemic hyperosmotic solutions and the administration of dexmedetomidine can increase GS permeability, thereby promoting drug transport to the CNS.^[^ ^147^ ^]^ External forces can also promote the function of the GS. For instance, transcranial near‐infrared light stimulation can enhance the flow of the brain's GS and increase the diameter of meningeal lymphatics by inducing vascular relaxation.^[^ ^177^, ^178^ ^]^

The discovery of the lymphatic system has opened up a new continent for CNS drug delivery, which is more conducive to drug penetration in the brain by circumventing the BBB. The drug delivery methods through the lymphatic system studied above have been validated accordingly and we have listed in Table 1 the advantages and disadvantages of different administration methods that circumvent BBB, the differences in the time and dosage for drug diffusion to lymph circulation and lesions, methods to improve administration efficiency, clinical research, and specific issues in clinical research. The time and dosage of drug diffusion into lymphatic circulation and lesions using different lymphatic administration methods depend on various factors such as individual physiological conditions, administration techniques, drug properties, and disease conditions. The choice of medication delivery method mainly depends on different CNS diseases that may require different drug delivery strategies. For example, for neurodegenerative diseases, a sustainable and long‐term drug delivery method should be chosen, such as intranasal or intrathecal administration, which can involve multiple small doses of drug delivery. For acute events such as stroke, rapid drug delivery may be required, such as brain parenchymal administration or intranasal administration. The urgency of treatment can also affect the choice of drug delivery strategies. In emergency situations, rapid‐acting drugs may be needed, while long‐term treatment may focus more on sustained drug release and stability. The research on MLV is currently still in the exploratory stage, and more methods are needed to evade phagocytes and target the location of CNS lesions.

**TABLE 1:** Differences in different administration methods.

Administration method	Advantage	Disadvantage	The differences in the time and dosage for drug diffuse to lymph circulation and lesions	Influence factor	Methods to improve administration efficiency	Clinical study	Clinical problems	References
Intraparenchymal administration	Directly acting on the brain Low drug loss The drug quickly reaches the brain	High cost Anesthesia and hospitalization required Slow drug diffusion Limited diffusion depth (1–3 mm) Unable to treat CNS communicable diseases Highly invasive and prone to complications	Drug diffusion is slow and depends on the physical and chemical properties of the drug	Properly place the sleeve The dosage form of the drug The distribution of drugs in tumors	Optimize drug delivery technology Improving drug bioavailability Using CED delivery	Delivery of neuroprotective or anti‐inflammatory drugs Treatment of neurodegenerative diseases Brain tumor treatment	Invasive and traumatic Limitations of drug dispersion High precision requirements Safety and toxicity of locally high‐concentration drugs Difficulty in efficacy evaluation	[20, 92, 96, 97, 102, 105, 179–184]
Intrathecal administration	Directly injected into CSF The only way to ensure the involvement of CNS targets	Highly invasive Easy to cause complications such as bleeding or infection Uneven distribution of drugs in brain parenchyma	Influenced by CSF flow and patient condition	Professional skills during the administration	Lipophilic drugs Continuous infusion using implantable drug delivery systems Whole body penetration method improves CSF flow into the brain	Targeting chronic cancer pain and chronic noncancer pain, such as spinal‐related pain, neuropathic pain, etc. Drug choices include opioid drugs (such as morphine and hydromorphone), local anesthetics (such as bupivacaine), α−2 receptor agonists (such as clonidine), and calcium channel blockers (such as ziconotide)	Adverse reactions Risk of infection during device implantation or replacement Drug dosage adjusted according to individualization Accurate positioning and operational techniques Possible complications such as CSF leakage Not all patients are suitable	[93, 138, 185–192]
Intranasal administration	Noninvasive Convenient Quickly target CNS by circumventing BBB Minimize systemic exposure to the greatest extent possible	Molecular size and properties are limited by nasal mucosal permeability Low dosage of self‐administered medication	Depending on the permeability of the nasal mucosa and the properties of the medication The nasal bioavailability of peptides and proteins is usually low Using liposome modification to increase the permeability of nasal mucosa	Drug properties Formulation factors Brain position Dosage and method of administration	Multiple small doses of medication Changing the performance of drugs	Mainly concentrated in neurodegenerative diseases, subarachnoid hemorrhage, stroke, and glioblastoma, among other brain diseases	Individual differences in nasal mucosal permeability Drug solubility (bioavailability) Safety and tolerability Accuracy of dose control More clinical trials are needed	[150, 152, 159, 160, 193–199]
Subcutaneous administration near CLN	Less invasive High drug delivery efficiency Less systemic side effects	Insufficient active targeting ability Easy to be engulfed by immune cells	Different experimental processes lead to different effects Time: Several hours Dosage: Several tens of times higher than intravenous injection	The properties and size of drugs and carriers	Increase drug targeting (liposomes) Inhibiting immune cell phagocytosis	Mouse experimental stage	Mouse experimental stage	[95, 165, 169, 170]
Intraosseous administration into the skull	Less invasive Allow for more precise drug targeting High brain drug concentration reduces systemic side effects	Currently, there is limited research Security and feasibility unknown Make the skull thinner The installation of drug delivery equipment may have potential disease symptoms	Different experimental processes lead to different effects Dosage: Several tens to hundreds of times higher than intravenous injection	Individual cranial differences Drug administration technology The physical and chemical properties of drugs	Optimizing drug formulations Precision drug delivery technology	Mouse experimental stage	Mouse experimental stage	[174, 175, 200]

## 4 BRAIN IMMUNITY

Due to the previous stereotype that there was no lymphatic system in the brain, people believed that missing the lymphatic system and BBB would block the transmission of immune cells, resulting in CNS's “immune privilege”.^[^ ^201^ ^]^ The discovery of the dural lymphatic system around the brain raised doubts about this concept.^[^ ^202^, ^203^, ^204^ ^]^ Research has shown that there are many types of immune cells present in the brain's meninges, providing immune monitoring for CNS.^[^ ^202^ ^]^ It was also found that B cells originate from the hematopoietic region of the skull and propagate from specialized vascular channels to the meninges, not entirely from the systemic circulation.^[^ ^205^ ^]^ The noninflammatory interleukin‐17 (IL‐17) of meningeal γδ T cells can promote the production of brain‐derived neurotrophic factors by glial cells, enhancing short‐term memory through glutamate synaptic plasticity.^[^ ^206^ ^]^ The IL‐4 produced by CD4^+^ αβ T cells in the meninges can also bias meningeal Mφ towards anti‐inflammatory properties and improve learning ability.^[^ ^207^ ^]^ When studying the meningeal T cell channel, studies found that the MLV in the meninges can also transport immune cells, and the CLN connected to them plays an important role in the transportation process. Studies have concluded that immune cells reside in specialized niches at their borders composed of brain boundaries, meninges, choroid plexus, and perivascular spaces. There are also micro channels among these components generating the interactions between the brain and the immune system. For example, the microchannels in the skull can supply immune cells to enter the brain, CSF enters the dura mater along the dural sinus and is discharged through lymphatic vessels, and the dural lymphatic vessels discharge extracellular fluid from the brain to the CLNs.^[^ ^171^, ^202^, ^204^, ^208^, ^209^ ^]^

The MLV is divided into dorsal MLV and basal MLV, which simultaneously provide a pathway for the CNS to connect to the CLN for clearing large molecules and transporting immune cells.^[^ ^210^ ^]^ Basal MLV in the skull clears large molecules from CSF to the lymphatic system through absorption and drainage, followed by clearance to CLN through dorsal MLV.^[^ ^211^ ^]^ At present, most experiments are based on dorsal MLV research, and some experiments have found that basal MLV can undergo remodeling and anti‐tumor immune effects; however, the effect is weaker compared to dorsal MLV.^[^ ^210^ ^]^ Experiments have shown that when CLN is removed, it could lead to an increase of T cells in the meninges, showing that the interaction between MLV and CLN jointly regulates the immune channels of CNS.^[^ ^30^ ^]^ Vascular endothelial growth factor (VEGF‐C) can activate the expansion of MLV and drive their generation and enhance the anti‐tumor activity of anti‐PD1 antibodies.^[^ ^212^ ^]^ At the same time, this anti‐tumor response can be eliminated by blocking CCL21/CCR7, indicating that CCL21/CCR7 is an important influencing factor in this response pathway.^[^ ^212^ ^]^ To verify that the expression of VEGF‐C can promote immune response in the MLV, Song et al.^[^ ^213^ ^]^ established a mouse model of glioblastoma and injected VEGF‐C into CSF and observed that the MLV was reshaped and the tumor was suppressed. Experimental data shows that VEGF‐C can promote the immune response of MLV when dCLN is intact. Compared to the immunogenicity of AAV vectors, mRNA vectors deliver VEGF‐C without being eliminated by immune cells, improving effective utilization and achieving better immune effects. When combined with checkpoint blockade therapy, glioblastoma can be completely eradicated. The main activity range of VEGF‐C injected into CSF is in the CSF and meninges while promoting the growth of LEC.^[^ ^213^ ^]^

**FIGURE 14:**
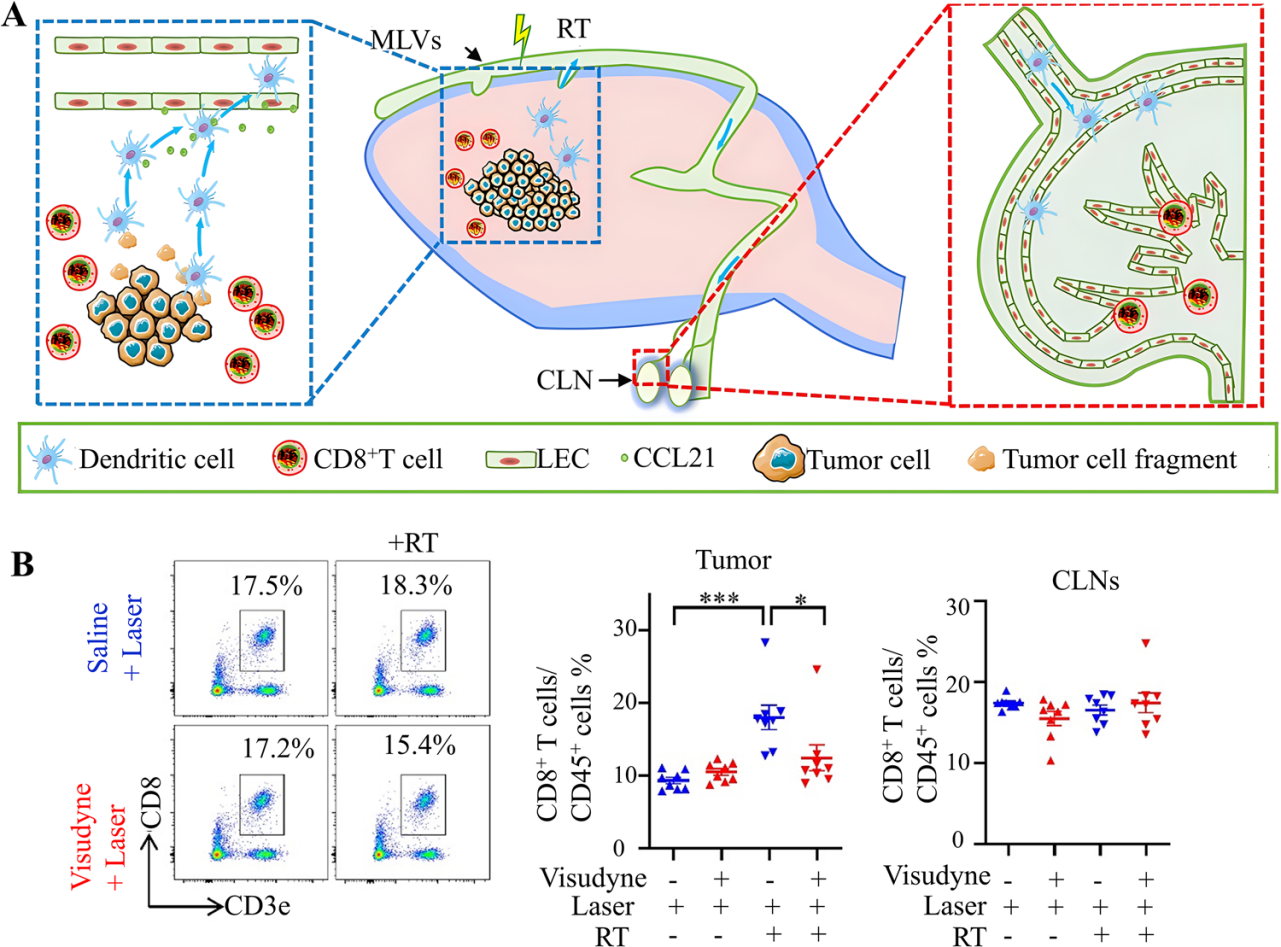
Immune regulation. (A) Functional schematic diagram of MLV and LN in RT anti‐tumor immunity. (B) The MLV‐deficient mouse model was established using a Visudyne laser. Flow cytometry images of CD8^+^T cells in CLN of different groups treated with Visudyne and RT, as well as quantitative representations of tumor and CLN (as a percentage of total CD45^+^cells) were obtained. Reproduced with permission.^[^ ^214^ ^]^ Copyright 2022, Springer Nature. MLV: meningeal lymphatic vessel, RT: radiotherapy, CLN: cervical lymph nodes, LEC: lymphatic endothelial cell.

Zhou et al.^[^ ^214^ ^]^ found that MLV not only performs immune regulation but also transports immune cells. They made bold associations between MLV and immune regulation during chemotherapy, confirming that MLV is involved in the immune response of RT (Figure 14A). The MLV connects CSF and CLN, hence CLN is an important pathway for CSF and immune cells to pass out of the brain. Zhou et al.^[^ ^214^ ^]^ first explored the impact of CLN on the anti‐tumor immunity of RT and established CLN removal mice as experimental groups. When CLN is absent, the tumor volume of mice significantly increases compared to RT alone. In addition, the percentage of immune cells significantly increased after RT, but decreased after CLN removal, indicating that CLN affects the anti‐tumor immune response of RT. Then, the effect of MLV on the anti‐tumor immunity of RT was studied. MLV‐deficient mice were also established, and the total number of CD8^+^T cells significantly decreased during MLV deficiency. When MLV is intact, the total number of DC cells significantly increases, further confirming the importance of MLV in immune response (Figure 14B). Zhou et al.^[^ ^214^ ^]^ further explored the role of VEGF‐C that promotes MLV generation in anti‐immunity and confirmed that overexpression of VEGF‐C enhances the anti‐tumor immune effect of RT through the CCL21 pathway.

Currently, several experimental studies on anti‐Aβ monoclonal drug (aducanumab) claimed to achieve Aβ immunotherapy in the treatment of Alzheimer's disease, and the crucial role of MLV in immunotherapy was emphasized in their experiment.^[^ ^52^ ^]^ If an individual's own MLV function is impaired, then the efficacy of Aβ immunotherapy will be significantly reduced.^[^ ^215^ ^]^ On the contrary, promoting the growth of MLV significantly improves the therapeutic effect, and its VEGF‐C may stimulate microglia to trigger higher IFN‐γ reactions.^[^ ^215^, ^216^ ^]^ The immune system of the brain does not work alone. Immune cells and lymphocytes circulate in the brain and have certain connections with the peripheral immune system, and the MLV are the main connecting points.^[^ ^210^ ^]^

## 5 OUTLOOK

The waste clearance pathway in the brain is very important; however, some macromolecular waste cannot be discharged through the blood circulation due to the presence of the BBB, resulting in accumulation in the brain. Many CNS diseases in the brain are caused by the accumulation of waste or toxic proteins in the brain, such as Aβ protein accumulation can damage the integrity of blood vessels and cause cerebral hemorrhage. Patients with Alzheimer's disease can observe Aβ protein accumulation and the accumulation of phosphorylated proteins is closely related to traumatic brain injury. The emergence of such GSs and meningeal lymphatic systems provides new pathways for brain clearance. There are multiple pathways for clearing wastes from the brain, which also provide multiple alternatives for drug delivery to the CNS. Due to the presence of the BBB, the entry of drugs into the CNS is greatly hindered. Therefore, utilizing the inclusiveness of the GS towards macromolecules may increase the transport efficiency of macromolecular drugs and drug carriers to the lesion site. Different injection methods may have different effects on drug delivery pathways. Intravenous drugs mainly overcome the BBB to reach the brain parenchyma. The use of CSF circulation to deliver drugs through GS circumventing the BBB has achieved positive outcomes. The GS provides very promising channels for the exchange of CSF and ISF in the brain parenchyma and can promote the function of the GS through factors such as arterial pulsation, sleep, and anesthesia, thereby promoting the flow of CSF into the brain parenchyma. The injection methods used for drugs to circumvent the BBB involve direct administration into brain parenchyma and CSF. The discovery of the meningeal lymphatic system stimulates new ideas for drug delivery to the brain parenchyma. Due to the connection between the MLV and the CLNs, drugs injected into the CLNs can reach CSF through the MLV, while also utilizing the GS to reach the brain parenchyma.

The performance of drugs and drug carriers is also a major consideration, such as molecular size, targeting, biological half‐life, biocompatibility, and avoidance of phagocytic cell phagocytosis. Additionally, the size of molecules is determined by factors such as the maximum size of substances absorbed by blood vessels, the intercellular spaces of MLV, and the gaps generated by astrocytes in the GS. The optimal size should be between the maximum size of substances absorbed by blood vessels and the gaps generated by astrocytes in the GS, which can avoid blood vessel absorption and enter the brain parenchyma through the GS. Carrying endogenous cells can also promote the targeting and delivery efficiency of drugs or drug carriers in the body. In the past few years, medical micro/nanorobots have attracted increasing interest in targeted drug delivery to the brain.^[^ ^217^, ^218^, ^219^ ^]^ Moreover, endogenous cells can be engineered into biohybrid microrobots to enhance their targeting ability and protect them from clearance by immunocytes.^[^ ^220^, ^221^, ^222^ ^]^ Medical micro/nanorobots are tiny machines that can respond to a variety of stimuli (such as chemicals, pH, light, heat, magnetic, electric, and ultrasound), move in complex physiological environment, and carry out diverse complicated medical tasks.^[^ ^223^, ^224^, ^225^, ^226^, ^227^, ^228^, ^229^, ^230^ ^]^ Furthermore, space travel is increasingly popular nowadays. However, many space stressors (such as microgravity and radiation) could result in severe health threats and pathophysiological changes to astronauts or space travelers.^[^ ^231^, ^232^, ^233^ ^]^ Typically, microgravity may cause many space diseases. In this context, exploring the performance of drugs or drug carriers under (simulated) microgravity plays a significant role in developing powerful therapies to protect the health of astronauts in space.^[^ ^234^, ^235^, ^236^, ^237^ ^]^

The modification of drugs is also a method to improve drug delivery efficiency. Polymers can be flexibly modified with specific ligands to enable nanoparticles to target tissues.^[^ ^238^ ^]^ The inherent bioactivity of polymers can control the activity of in vivo transport systems, offering greater stability and higher encapsulation efficiency compared to other nanocarriers.^[^ ^239^ ^]^ They can also exhibit different shapes, such as micelles and dendrimers, with sizes and structures similar to natural carriers.^[^ ^240^ ^]^ The increased size of dendritic macromolecules can improve lymphatic absorption efficiency, and enhancing hydrophobicity can also increase retention in the lymph nodes.^[^ ^241^ ^]^ When PEGylation is used to increase the size and hydrophilicity of dendritic macromolecules, it can improve lymphatic drainage.^[^ ^242^ ^]^ Cell‐penetrating peptides, as drug delivery carriers, can interact with the protein glycocalyx layer on the cell membrane surface and translocate into cells, thereby enhancing cell permeability and promoting cellular uptake and absorption of drugs.^[^ ^243^, ^244^, ^245^ ^]^ However, they have drawbacks, such as a lack of cell‐penetrating specificity, which can lead to the widespread distribution of drugs.^[^ ^246^ ^]^ Therefore, how to accurately apply cell‐penetrating peptides is also a significant challenge. Nanomaterials have achieved considerable advancements in the field of drug delivery, yet the issue of how to clear them from the brain remains a problem that needs to be addressed clinically. Research indicates that nanoparticles are not only capable of exiting the brain through the paravascular pathways of the GS but can also be actively transported to the BBB via exosomes. Microglia cells can collect nanoparticles and pass them on to the lymphatic drainage or the BBB for departure from the brain.^[^ ^247^ ^]^ This represents a significant breakthrough for resolving the metabolic challenges of future nano‐delivery systems.

The current research explores novel drug delivery methods that circumvent BBB and the importance of the brain lymphatic system in drug delivery. Patients with different CNS diseases have completely different states of brain lymphatic system, neurotransmitters, and neural cell metabolism, so there are also different administration efficiencies for the same administration method. Similarly, the function of the brain lymphatic system to clear brain waste can also affect the retention time of drugs. It is important to maintain the drug's better efficacy at the lesion site and monitor the entire treatment process. Preliminary experiments have been conducted on subcutaneous administration near CLN and intraosseous administration into the skull in animal models, and good results have been achieved. However, it is difficult for animal models to simulate and replicate the complexity of human CNS diseases. More possibilities for preclinical research are essential. Sometimes drugs may not need to be delivered to the brain and directly act to maintain the function of the lymphatic system and meningeal lymphatic system, which may treat some CNS diseases. The development of drugs may also extend from treating neurological diseases to preventing CNS diseases. In the future, we should focus more on the impact of neurochemistry on drug delivery, exploring the best drug delivery methods that are more suitable for each disease and strategies to promote drug delivery efficiency in neurochemistry from a more fundamental research perspective.

## CONFLICT OF INTEREST STATEMENT

The authors declare no conflicts of interest.
